# 2-Arachidonoylglycerol Modulates CXCL12-Mediated Chemotaxis in Mantle Cell Lymphoma and Chronic Lymphocytic Leukemia

**DOI:** 10.3390/cancers15051585

**Published:** 2023-03-03

**Authors:** Magali Merrien, Agata M. Wasik, Christopher M. Melén, Mohammad Hamdy Abdelrazak Morsy, Kristina Sonnevi, Henna-Riikka Junlén, Birger Christensson, Björn E. Wahlin, Birgitta Sander

**Affiliations:** 1 Division of Pathology, Department of Laboratory Medicine, Karolinska Institutet, 171 77 Stockholm, Sweden; 2 Division of Haematology, Department of Medicine at Huddinge, Karolinska Institutet, 171 77 Stockholm, Sweden; 3 Unit of Haematology, Karolinska University Hospital, 171 76 Stockholm, Sweden; 4 Pathology and Cancer, Karolinska University Hospital, 141 86 Stockholm, Sweden

**Keywords:** leukemia, lymphoma, cannabinoid receptors, 2-arachidonolglycerol, chemotaxis, CXCR4

## Abstract

**Simple Summary:**

Most lymphoma patients relapse after therapy, mainly due to malignant cells hidden in tissues and protected by different components from the surrounding microenvironment. In this study, we describe the interaction between two important factors from the microenvironment, the biolipid 2-arachidonoylglycerol and the chemokine CXCL12. We specifically aimed at better understanding the role of 2-arachidonoylglycerol in the migration of lymphoma cells, and how it affects the migration towards CXCL12 and specific downstream signaling pathways. 2-arachidonoylglycerol signals through the cannabinoid receptors type 1 and type 2 (CB1 and CB2). CB1 and CB2 are variably expressed in B-cell lymphoma. We demonstrate that 2-arachidonoylglycerol induces migration of B-lymphoma cells via CB1 and CB2. We also provide evidence that the signaling pathways activated by 2-arachidonolglycerol and CXCL12 are cross-talking.

**Abstract:**

To survive chemotherapy, lymphoma cells can relocate to protective niches where they receive support from the non-malignant cells. The biolipid 2-arachidonoylglycerol (2-AG), an agonist for the cannabinoid receptors CB1 and CB2, is released by stromal cells in the bone marrow. To investigate the role of 2-AG in lymphoma, we analyzed the chemotactic response of primary B-cell lymphoma cells enriched from peripheral blood of twenty-two chronic lymphocytic leukemia (CLL) and five mantle cell lymphoma (MCL) patients towards 2-AG alone and/or to the chemokine CXCL12. The expression of cannabinoid receptors was quantified using qPCR and the protein levels visualized by immunofluorescence and Western blot. Surface expression of CXCR4, the main cognate receptor to CXCL12, was analyzed by flow cytometry. Phosphorylation of key downstream signaling pathways activated by 2-AG and CXCL12 were measured by Western blot in three MCL cell lines and two primary CLL samples. We report that 2-AG induces chemotaxis in 80% of the primary samples, as well as 2/3 MCL cell lines. 2-AG induced in a dose-dependent manner, the migration of JeKo-1 cell line via CB1 and CB2. 2-AG affected the CXCL12-mediated chemotaxis without impacting the expression or internalization of CXCR4. We further show that 2-AG modulated p38 and p44/42 MAPK activation. Our results suggest that 2-AG has a previously unrecognized role in the mobilization of lymphoma cells by effecting the CXCL12-induced migration and the CXCR4 signaling pathways, however, with different effects in MCL compared to CLL.

## 1. Introduction

B-cell lymphomas are caused by genetic aberrations in the lymphoma cells, but the tumor tissue microenvironment contributes to the growth and survival of lymphomas, such as mantle cell lymphoma (MCL) and chronic lymphocytic leukemia (CLL) [1,2], two mature B-cell lymphoma types that have similar mechanisms for interacting with the microenvironment [3]. The microenvironment influences treatment response and drug resistance, especially in the context of the B-cell receptor (BCR) signaling inhibitors [4]. Both MCL and CLL are dependent on survival signaling provided by the BCR, which is a B-cell specific surface immunoglobulin complex that recognizes antigens. In MCL and CLL, the BCR signaling is continuously active and over-stimulated. Inhibition of the BCR downstream signaling with the Bruton’s tyrosine kinase (BTK) inhibitors Ibrutinib or Acalabrutinib, results in egress of malignant cells from the lymph nodes to the blood circulation. However, many patients have to stop therapy due to side effects or non-responsiveness as the lymphoma cells acquire mutations to escape the drug [5]. Further knowledge on mechanisms involved in the supportive lymphoid microenvironment is needed, both to understand lymphoma biology and to provide novel potential treatment strategies.

Recent studies in normal mature B cells and in CLL have provided evidence for crosstalk between the signaling pathways of BCR and the G-protein coupled receptor CXCR4 [6,7,8,9,10]. CXCR4, highly expressed in MCL and CLL [1,11], is a crucial chemokine receptor regulating B-cell homing to lymph nodes and bone marrow. It is activated by the chemokine CXCL12, which is secreted by stromal cells in these compartments [12,13,14]. CXCL12 is therefore involved in communication between the microenvironment and malignant lymphocytes and promotes cell survival, metastasis and chemotherapy treatment resistance through CXCR4 signaling [15].

Cannabinoid receptors are also G-protein coupled receptors and are highly expressed in most cases of MCL compared to non-malignant mantle zone B-cells and reactive lymph nodes [16,17], and in approximately half of CLL cases [18,19]. CB1 (encoded by *CNR1*) is highly expressed in the neurons in CNS and regulates synaptic signaling while expression of CB2 (encoded by *CNR2*) is high in peripheral tissues and immune cells. In mice, CB2 is involved in the formation of the splenic marginal zone [20,21] and in the retention of immature B cells in bone marrow sinusoids [22]. CB2 has been suggested to control glucose intake and energy supply in malignant B cells [23]. The cannabinoid receptors are part of the endocannabinoid system, together with the endogenous agonists called endocannabinoids and the enzymes that synthetize, transport and degrade endocannabinoids. The endocannabinoid system is dysregulated in many types of cancers [24], and one of the main endocannabinoids, 2-arachidonoylglycerol (2-AG), was found to be elevated in both mouse cancer models and human cancer samples, including solid tumors and hematological malignancies [25,26]. 2-AG is normally produced on demand by mesenchymal stromal cells in bone marrow [27], and the reports from Sailler et al. and Zhang et al. describe high 2-AG levels being related to tumor progression and metastasis [25,26]. In addition, the dysregulated expression pattern of the enzymes responsible for the synthesis and degradation of endocannabinoids in MCL suggests an accumulation of endocannabinoids and, hence, an enhanced cannabinoid receptor signaling [28].

Although the applicability of targeting the cannabinoid receptors for therapy in lymphoma is yet to be verified [18,23,29,30,31], the potential physiological impact of high cannabinoid receptors expression in MCL and CLL needs investigation. We previously reported an inverse correlation between CB1 mRNA levels and lymphocytosis in MCL patients [28], and more recently, Clot et al. reported that *CNR1* is downregulated in leukemic non-nodal MCL compared with conventional nodal MCL [32]. Therefore, we hypothesize that the cannabinoid receptors regulate homing, tissue retention and/or egress of the malignant B-cells from to the bloodstream in response to endocannabinoids.

To better understand the role of the endocannabinoid system in lymphoid malignancies, we analyzed how 2-AG regulates the chemotaxis of lymphoma cells and investigated its ability to modify the response to CXCL12 effect on cell migration in MCL and CLL.

## 2. Materials and Methods

### 2.1. Cell Lines and Primary Samples

The MCL cell lines JeKo-1, Granta519, JVM-2 and the T-cell line Jurkat were obtained from DSMZ (Braunschweig, Germany). All cell lines were maintained in culture in RPMI 1640 medium, GlutaMAX supplement, and HEPES (RPMI-GM, Gibco, UK), supplemented with 50 µg/mL of gentamicin and 10% fetal bovine serum (FBS) (Gibco) under the conditions of 5% CO_2_ at 37 °C. The glioblastoma cell line U-251 was purchased from ECACC and was kept in culture in MEM complete medium supplemented with 10% FBS (Sigma-Aldrich/Merck, Darmstadt, Germany) at 5% CO_2_ at 37 °C, until confluency before splitting them using trypsin-EDTA (0.25%, ThermoFisher Scientific, Paisley, UK) to detach the cells. MCL and CLL blood samples were collected at Karolinska University Hospital, Huddinge. The study was approved by the Regional Central Ethical Review Board at Karolinska Institutet and patients gave informed consent, in compliance with the Declaration of Helsinki. 

### 2.2. Lymphoma Cell Isolation from Peripheral Blood Samples

Blood samples were enriched for B cells by negative selection using RosetteSep™ containing a cocktail of antibodies (StemCell Technologies, Saint Egreve, France) according to the manufacturer’s protocol. Thereafter, cells were collected using Ficoll-Paque PLUS (GE Healthcare Life Science, Chicago, IL, USA), washed with PBS and directly used for functional assays or viability frozen in 50% RPMI-GM, 40% FBS, 10% DMSO, and preserved at −150 °C. After B cell enrichment by RosetteSep™, the lymphoma cell purity (68.4–99.8%; median 97.1%, Supplemental Table S1) was assessed by double expression of CD19 and CD5 within the CD45 positive population (Supplemental Figure S1A), using flow cytometry.

### 2.3. Antibodies and Reagents

Brilliant violet 421 anti-human CXCR4, allophycocyanin anti-human CD5 and phycoerythrin anti-human CD19 antibodies were obtained from BioLegend, BD Biosciences and Dako, respectively. Antibodies used for Western blotting: anti-phospho ERK1/2 (Thr202/Tyr204), anti-total ERK1/2, anti-phospho p38 (Thr180/Tyr182), anti-total p38, anti-phospho Akt (Ser473) and anti-total Akt were all from Cell Signaling Technology (Danvers, MA, USA). Antibody anti-CB1 was purchased from ThermoFisher (cat#PA1-743), and anti-CB2 was from Santa-Cruz (cat#293188). CXCL12 was from R&D Systems (biotechne, Abingdon, UK), 2-arachidonoylglycerol was from Tocris Bioscience and Calcein-AM was purchased from ThermoFisher Scientific. The CB1 antagonist AM6545 was purchased from Tocris Bioscience and the CB1 inverse agonist SR141716 and the CB2 antagonist SR144528 were from Cayman Chemical. The CXCR4 antagonist AMD3100 was purchased from Sigma.

### 2.4. siRNA Introduction by Electroporation

Transient downregulation of the *CNR1* and *CNR2* genes was obtained using small interference RNA (siRNA). JeKo-1 cells were transfected with commercial gene-specific siRNAs (*CNR1*: s3261; *CNR2*: s3265 and s3263; ThermoFisher) or negative siRNA by electroporation method using the AMAXA machine and the Nucleofector Kit C (Lonza, Basel, Switzerland). Briefly, 1 µM of the siRNA was introduced together with the Nucleofector solution, the program X-01 from the electroporation machine was applied, 20% FBS in RPMI-GM was added to the cells immediately after transfection and then the cells were transferred to a 6-well plate containing 10% FBS in RPMI-GM and incubated at 37 °C, 5% CO_2_ until functional assay. 

### 2.5. RNA Isolation and cDNA Synthesis

RNA isolation was performed by RNeasy Plus Mini Kit (Qiagen, Hilden, Germany) according to manufacturer’s protocol. The amount of mRNA was measured with the NanoDrop ND-1000 spectrophotometer (ThermoFisher Scientific). Complementary DNA (cDNA) was synthetized using Omniscript Reverse Transcription Kit (Qiagen) according to manufacturer’s protocol. RNaseOut recombinant ribonuclease inhibitor and the oligo dT primers were purchased from Invitrogen (Waltham, MA, USA).

### 2.6. qPCR

mRNA expression levels of genes encoding for CB1 (*CNR1*) and CB2 (*CNR2*) were assessed by real-time quantitative PCR using Platinum SYBR Green qPCR Supermix-UDG (Invitrogen). Custom-made primers were purchased from Invitrogen, and sequences for respective genes were as follows: *CNR1* forward: 5′-CATTAAGACGGTGTTTGCATTCT-3′, reverse: 5′-CGTGTCGCAGGTCCTTACTC-3′; *CNR2* forward: 5′-GACACGGACCCCTTTTTGCT-3′, reverse: 5′-CCTCGTGGCCCTACCTATCC-3′; β-actin (*ACTB*) forward: 5′-AAAGACCTGTACGCCAACACA-3′, reverse: 5′-AGTACTTGCGCTCAGGAGGA-3′. Since false positive signals may appear during the quantification of *CNR2* after introduction of specific siRNA [33], we used a different set of primers to quantify *CNR2* expression after silencing only: *CNR2*_2 forward: 5′-ATGAGGCCTCTTCCCAATTT-3′; *CNR2*_2 reverse: 5′-CAGGCTGTCTTCCAGGAGTC-3′. Each sample was prepared in triplicates in a 96-well plate (BioRad, Leipzig, Germany) and the reactions were performed with the C1000 Thermal Cycler (BioRad). An initial denaturation step was performed at 95 °C for 3 min, followed by 15 s at 95 °C, then by 30 s 57 °C step to allow primer annealing and elongation. The two latest steps were repeated 39 times for the amplification. The results were analyzed, and cycle threshold (Ct) values of transcripts were quantified using CFX manager software (BioRad). ∆Ct values (meaning that a low value corresponds to a high expression, a high value to a low expression, and a level at 0 to no expression) were determined using ACTB as reference. ∆Ct value from either the control condition or from enriched B cells from a buffy coat was used to calculate ∆∆Ct values and the change in gene expression levels was determined by relative fold increase (RFI; 2^−∆∆Ct^).

### 2.7. Activation of p38, AKT and p44/42 Signaling Pathway and Western Blot

Cells were rested for 1 to 3 h in 0.1% fatty acid free bovine serum albumin (BSA, Sigma-Aldrich)/RPMI-GM at 37 °C, then resuspended in 0.1% BSA/RPMI-GM at 2 × 10^6^ cells per mL, and treated with CXCL12 (200 ng/mL) and/or 2-AG (100 nM) for 2 min. AMD3100 (25 µg/mL), SR141716 (10 nM), SR144528 (10 nM), AM6545 (10 nM), LY294002 (50 µM), SB202190 (25µM) and PD98059 (50µM) pre-treatments were done for 20 min to 1 h prior to 2-AG +/− CXCL12 treatments. Cells were washed with cold PBS to stop the reaction, pelleted, placed on ice and cell lysates were prepared using RIPA buffer supplemented with phosphatase and protease inhibitors cocktails (all from Sigma-Aldrich). For each specific pathway, proteins were resolved on two parallel 12% NuPAGE gels (Invitrogen) (one for phospho-protein, one for the corresponding total protein) and transferred using a semi-dry transfer system onto PVDF membranes (Millipore, Burlington, MA, USA). Non-specific binding sites were blocked with 10% BSA TBS-T solution for 1 h at room temperature (RT), then probed overnight at 4 °C with the respective primary antibodies: anti-phospho-AKT (S473), anti-phospho-p44/42 (T202/Y204), anti-phospho-p38 (T180/Y182), total-AKT, total-p44/42, total-p38 or anti-GAPDH (all from Cell Signaling) in 5% BSA in TBS-T. Membranes were then washed in TBS-T and probed with secondary antibodies (HRP-conjugated anti-rabbit or anti-mouse; GE Healthcare). Blots were developed using Western Lightning Plus ECL (PerkinElmer, Groningen, The Netherlands) and visualized using LiCor machine. For re-probing of membranes, HRP was blocked using the SG substrate kit (Vector Laboratories, Newark, CA, USA). Bands intensity was measured using ImageJ software. The quantification was done by first normalizing bands intensities for total or phospho to their respective GAPDH control on the respective blot. Then, we could normalize phospho/GAPDH to total/GAPDH, giving the activation status for each pathway. The same Western blot protocol was used for detection of cannabinoid receptors. 

### 2.8. Flow Cytometric Analysis of CXCR4 Surface Expression

Basal CXCR4 surface expression was analyzed in MCL cell lines and in primary MCL and CLL cells. JeKo-1 cell line or freshly isolated cells were treated with CXCL12 (200 ng/mL) and/or 2-AG (100 nM) for 10 min or 4 h, washed twice with ice-cold PBS and stained with IgG control or anti-CXCR4 antibody for 20 min on ice. Stained cells were analysed by BD FACSCanto II (BD Biosciences, Erembodegem, Belgium). Data analysis was conducted using FlowJo for Windows version 10.

### 2.9. Immunofluorescence Staining

For immunofluorescence staining, cytospins from MCL cells in culture were prepared (2–5 × 10^4^ cells/slide) or the cell line U-251 was cultured on an 8-well slide (2 × 10^4^ cells/well) for 48 h prior to the staining protocol. Cells were first fixed with 4% paraformaldehyde for 10 min at RT. After two washes in PBS-T, permeabilization was achieved with 0.5% TritonX100 for 10 min at RT. Unspecific sites were then blocked with 4% donkey serum for 1 h at 37 °C, before adding the primary antibody anti-CB1 (rabbit) or anti-CB2 (mouse), which was incubated for 2 h at RT in humidity chambers. Respective secondary antibodies (donkey anti-mouse coupled to FITC or donkey anti-rabbit coupled to TRITC) were applied for 1 h at RT after washes with PBS-T. Excess of antibodies was washed with PBS-T and slides were dried before mounting the coverslip with mounting medium containing DAPI. Slides were then visualized under a confocal inverted microscope Nikon Eclipse Ti-E, A1R, using X20 magnification objective. Cell lines U-251 and Jurkat were used as positive controls for CB1 and CB2 staining, respectively (Supplementary Figure S5). Analysis of the images obtained from ICC/IF was performed using CellProfiler (version 4.07), Broad Institute of MIT and Harvard, USA. Pipelines were created in CellProfiler to determine mean fluorescence intensity of CB1 (TRITC) and CB2 (FITC) immunofluorescence staining per cell. Either CB1 or CB2 were assigned as child objects to DAPI-stained nuclei which were used as surrogates of total cell counts. Percentage of CB1+ or CB2+ cells were calculated by dividing the number of fluorescently labelled cells by the total number of cells per slide. (Pipelines used for image analysis are uploaded in Supplementary Files).

### 2.10. Chemotaxis

Chemotaxis experiments were performed using a Boyden chamber-based method in which inserts shield the fluorescence in the upper chamber and thus allow detection of only migrated fluorescently labelled cells (FluoroBlok technology, Corning, Durham, NC, USA). FluoroBlok™ inserts were used for MCL cell lines in 8 µm pore-size, in 24-well plates with fluorescent cell number as the readout using Nikon Eclipse Ti confocal microscopy system, or in 96-well plate format with fluorescence intensity readout using spectrofluorometer (FLx800, BioTek, Winooski, VT, USA), which corresponded to the cell number (see Supplementary Figure S2A). Both settings were used for JeKo-1 cell line and the latter would be used for Granta519 and JVM-2 since they form aggregates making it difficult to quantitate migrated cells by microscopy. In the case of primary MCL and CLL cells, 5 µm inserts were used (Costar, Washington, DC, USA), in 24-well plates using fluorescent cell number by microscopy as the read out. Before the chemotaxis assay, cultured cells were washed in order to remove FBS. Frozen isolated primary cells were thawed and kept in 10% FBS in RPMI-1640-GlutaMAX for 1 h. Primary and cultured cells were then stained with the cell-permeable green fluorescent dye calcein-AM (1 µM) and placed at the top on the inserts. The bottom chamber contained RPMI-1640-GlutaMAX medium supplemented with 0.1% fatty acid free BSA, and/or CXCL12 (200 ng/mL) and/or 2-AG (100 nM). Since 2-AG was dissolved in DMSO, DMSO was used as vehicle control at the same final concentration. When using the 24-well plates and confocal microscopy as the read out, cells were allowed to migrate for 6 h, images of the bottom well in focus were captured every 30 min and fluorescent cells were counted using NIS-Elements AR software. When using 96-well plates and fluorescence intensity as the read out, cells were allowed to migrate for 4 h and fluorescence at 485/528 nm was measured at that time point.

### 2.11. Statistics

Analyses were performed using GraphPad Prism 8.3.0. Paired sample t, Mann–Whitney, Wilcoxon signed-rank, Friedman for multiple comparison and Spearman tests were used. *p* < 0.05 was considered significant.

## 3. Results

### 3.1. Chemotaxis of Primary MCL and CLL Cells towards 2-AG

Lymphoma cells isolated from blood of patients with MCL (n = 5) and CLL (n = 22) were subjected to a chemotaxis assay. The clinical characteristics of the patients are described in Table 1.

**Figure 1:**
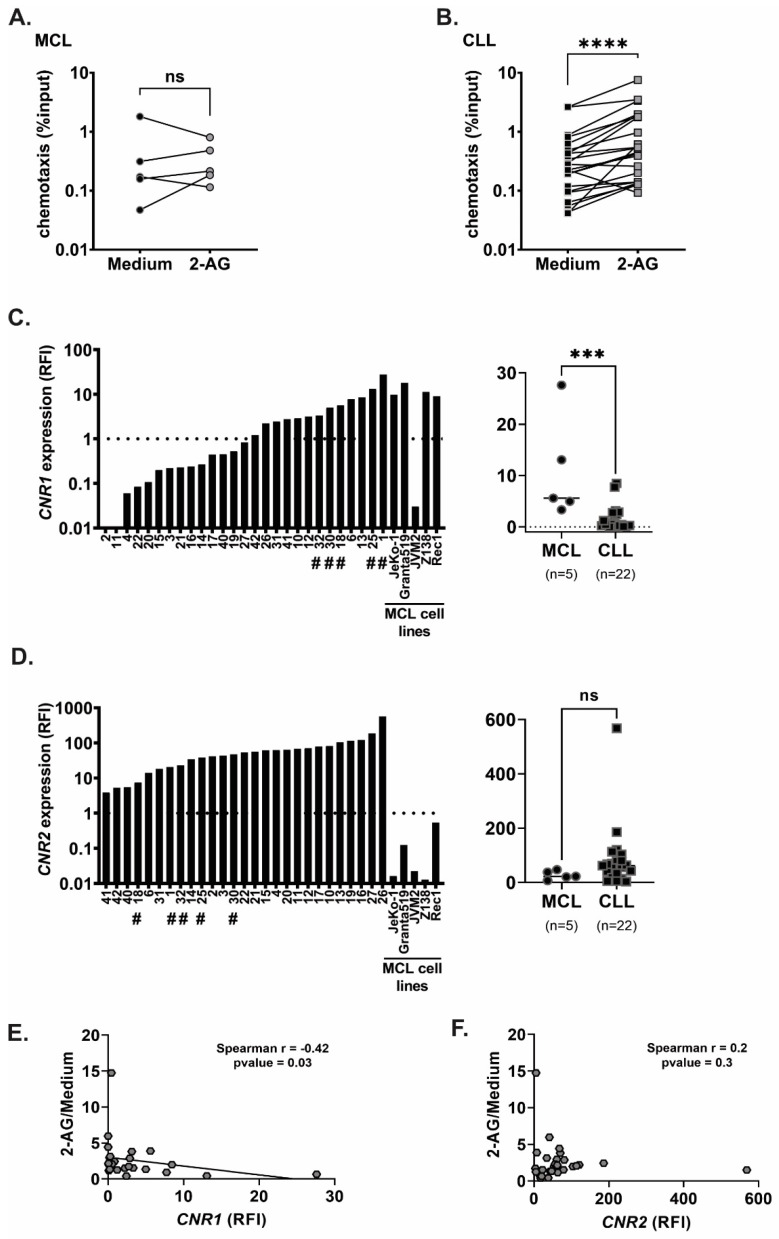
Chemotaxis towards 2-AG and cannabinoid receptor expression in MCL and CLL. (**A**) MCL (n = 5) and (**B**) CLL (n = 22) cells were subjected to chemotaxis towards vehicle or 2-AG (100 nM), number of migrated cells is shown as percentage of input (log10 scale) at 4 h time point, line represents paired samples; Wilcoxon matched-pairs signed rank test, ns: non-significant, **** *p* < 0.0001. (**C**) *CNR1* and (**D**) *CNR2* expression shown as relative fold increase (RFI) in MCL (distinguishable by #) and CLL cells, in the bar graphs and in MCL cell lines, the dotted line represents the expression in normal B cells from buffy coat taken as reference in the qPCR; Mann–Whitney test was performed to compare expression between MCL and CLL samples, ns: non-significant, *** *p* < 0.001. (**E**,**F**) Correlation between chemotaxis towards 2-AG (relative to medium) and (**E**) *CNR1,* or (**F**) *CNR2* expression in MCL and CLL primary samples combined with MCL cell lines; Spearman correlation test.

Fluorescently labelled lymphoma cells isolated from MCL (Figure 1A) and CLL (Figure 1B) were allowed to migrate for 4 h towards either vehicle or towards the endocannabinoid 2-AG used at the physiological concentrations found in human plasma (100 nM) [34]. Twenty out of twenty-two CLL samples (91%) migrated towards 2-AG with a range of 1.2–15-fold increase chemotaxis towards 2-AG compared to chemotaxis towards medium alone (median of 2.2 fold; *p* < 0.0001, Figure 1B). In MCL, 2-AG enhanced the chemotaxis of three out of five cases, with an increase of 1.4- to 3.9-fold compared to medium (median of 1.5 fold; *p* > 0.05, ns) (Figure 1A).

### 3.2. Cannabinoid Receptors Are Differentially Expressed in MCL and CLL

Because 2-AG is agonist to both cannabinoid receptors, we analyzed *CNR1* and *CNR2* expression in all samples (Figure 1C,D). All samples but two (both from CLL patients) expressed *CNR1* above the detection level. Six out of nineteen CLL had a higher *CNR1* expression than normal B cells from buffy coat (median of 0.4 relative fold increase; range 0.06–8.5), while all MCL samples expressed *CNR1* at higher levels than normal B cells (median of 5.6 relative fold increase; range 3.3–27.6), which was significantly higher than in CLL cells (*p* = 0.0007). *CNR2* was expressed in all patients’ samples, with a high range from 3.8 to 568.4 relative fold increased (median of 53.8). *CNR2* was not expressed significantly higher in CLL cells compared to MCL cells despite the different median of 61.6 and 22.9, respectively (*p* = 0.08). Considering that the chemotaxis pattern towards 2-AG was also different between MCL and CLL samples, we hypothesized that this could be due to the different expression of the cannabinoid receptors. Correlation analysis showed a moderate but significant inverse correlation between the *CNR1* levels and chemotaxis towards 2-AG when data from CLL and MCL were combined (Figure 1E; r = −0.42; *p* = 0.03). Thus, high *CNR1* expression was associated with lower migration towards 2-AG. Such correlation was not found with *CNR2* gene expression (Figure 1F). These results indicate involvement of CB1 in 2-AG mediated chemotaxis in lymphoma.

### 3.3. Both CB1 and CB2 Receptors Contribute to the 2-AG Mediated Chemotaxis in MCL Cell Line

**Figure 2:**
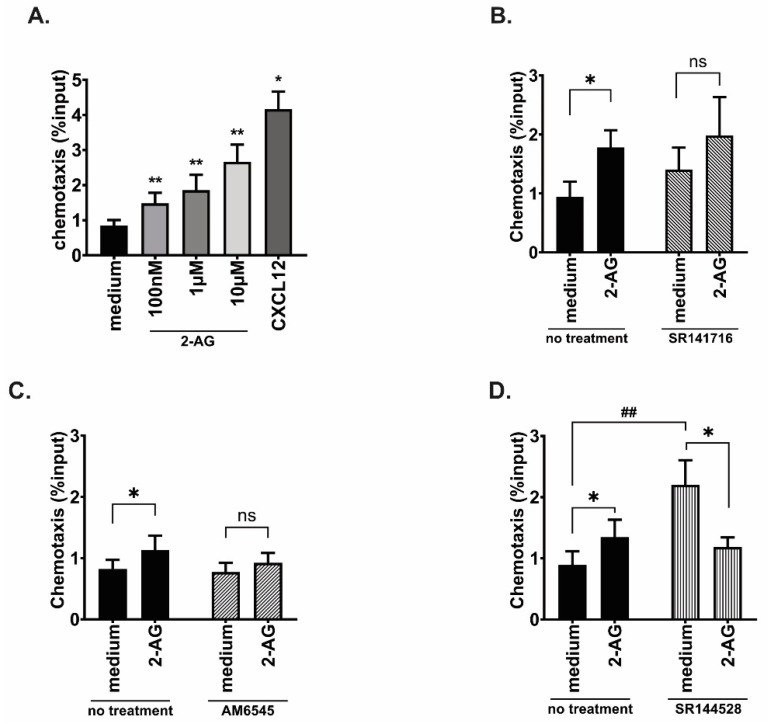
2-AG-mediated chemotaxis in MCL cell line JeKo-1. (**A**) JeKo-1 cells were subjected to chemotaxis towards vehicle or 2-AG at three different concentrations: 100 nM, 1µM and 10µM, or CXCL12 (200 ng/mL) as a positive control of chemotaxis, bars represent average of seven experiments and error bars are standard deviation; Paired *t*-test, * *p* < 0.05, ** *p* < 0.01. (**B**–**D**) Chemotaxis assays towards vehicle or 2-AG (100 nM) after JeKo-1 cells were incubated for 20 min with the CB1 antagonists (**B**) SR141716 (10 nM; n = 4), (**C**) AM6545 (10 nM; n = 5), or (**D**) with the CB2 inverse agonist SR144528 (10 nM; n = 8); Paired *t*-test, * *p* < 0.05, ns: non-significant, Wilcoxon matched-pairs signed rank test, ## *p* < 0.01.

In order to investigate via which cannabinoid receptor 2-AG was inducing chemotaxis, we used, as a model, the JeKo-1 MCL cell line, which expresses both *CNR1* and *CNR2*. First, we verified that JeKo-1 cells could migrate towards 2-AG (Figure 2A). After 4 h, the cells had a significantly increased chemotaxis index towards the same concentration of 2-AG as the primary cells (100 nM; 1.7-fold increase compared with medium alone, *p* = 0.03). The 2-AG induced chemotaxis was concentration dependent (Figure 2A). To elucidate whether CB1 or CB2 was mediating the migratory response to 2-AG we used two approaches: silencing the genes and targeting receptors with specific inhibitors. For silencing we used gene-specific siRNAs, then verified the efficacy of silencing by quantifying the mRNA expression with qPCR (Supplementary Figure S2B). After siRNA for *CNR1*, *CNR1* was downregulated by 75% at 24 h and by 62% after 48 h. *CNR2* downregulation was only 60% after 24 h and the levels were back to the basal levels at 48 h. Viability was not affected by the *CNR*-specific siRNAs (Supplementary Figure S2C). However, at the 48 h time point, downregulating *CNR2* gene significantly increased *CNR1* expression (*p* = 0.02). We performed chemotaxis assay towards 2-AG with the transfected cells and could see a tendency towards an inhibition of chemotaxis towards 2-AG after downregulation of *CNR1* and *CNR2* genes separately (Supplementary Figure S2D). However, these changes were not significant, perhaps due to the potential effect of gene cross-regulation. 

We therefore decided to use specific antagonists for CB1 (AM6545 and SR171416, both at 10 nM) and a CB2 inverse agonist (SR144528, 10 nM). After treatment with the CB1 antagonist SR171416 or AM6545, chemotaxis towards 2-AG was not any more significantly different from chemotaxis towards medium at 4 h (*p* = 0.2 and *p* = 0.14, respectively; Figure 2B,C). After treating the cells with CB2-specific inverse agonist SR144528, the chemotaxis towards 2-AG was reduced by 50% compared with chemotaxis towards medium alone (*p* = 0.04; Figure 2D). However, it is important to note that SR144528 treatment increased chemotaxis towards medium alone compared with untreated cells (*p* = 0.008, Figure 2D).

### 3.4. Chemotaxis towards 2-AG and CXCL12 in Combination

**Figure 3:**
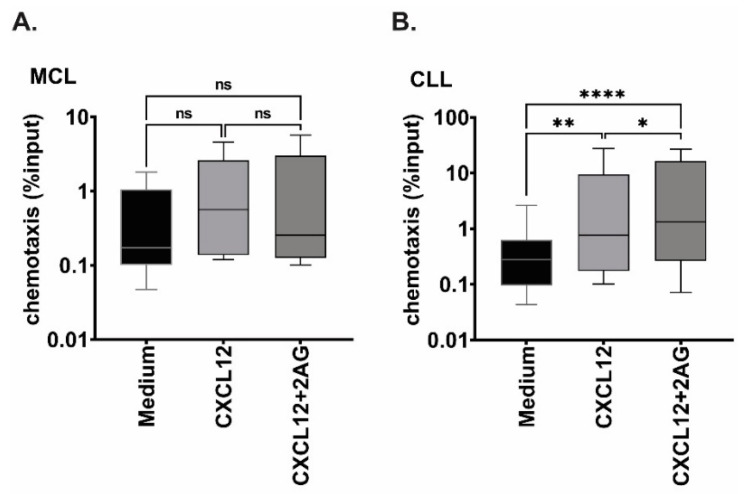
Effect of 2-AG on CXCL12-mediated chemotaxis. (**A**) MCL (n = 5) and (**B**) CLL (n = 22) cells were subjected to chemotaxis towards CXCL12 (200 ng/mL) or the combination of 2-AG (100 nM) and CXCL12 (200 ng/mL), number of migrated cells is shown as percentage of input (log10 scale) at 4 h; Friedman test for multiple comparison, ns: non-significant, * *p* < 0.05, ** *p* < 0.005, **** *p* < 0.0001.

CXCL12 and its receptor CXCR4 are among the main factors regulating lymphoma cell trafficking from blood to lymphoid tissue. Because 2-AG is also present within the microenvironment, we wondered whether 2-AG could modulate CXCL12-induced chemotaxis. We subjected MCL and CLL primary cells to chemotaxis assay towards CXCL12 alone or towards CXCL12 in combination with 2-AG (Figure 3A,B, respectively). CXCL12 alone increased chemotaxis of all samples but two (1 MCL and 1 CLL) from 1.4- to 95-fold increase compared with medium (median of 3.5-fold increase, *p* < 0.01). However, when CXCL12 was combined with 2-AG in the bottom chamber, MCL and CLL cells behaved differently. In CLL, the combination CXCL12 + 2-AG increased migration compared with CXCL12 alone (median of 4.0-fold increase; *p* = 0.015, Figure 3B), while in MCL 3/5 samples had a reduced chemotaxis when CXCL12 was combined with 2-AG in bottom chamber (up to 50% reduction, Figure 3A). 

### 3.5. Effect of 2-AG on CXCL12-Mediated Chemotaxis in MCL Cell Lines

**Figure 4:**
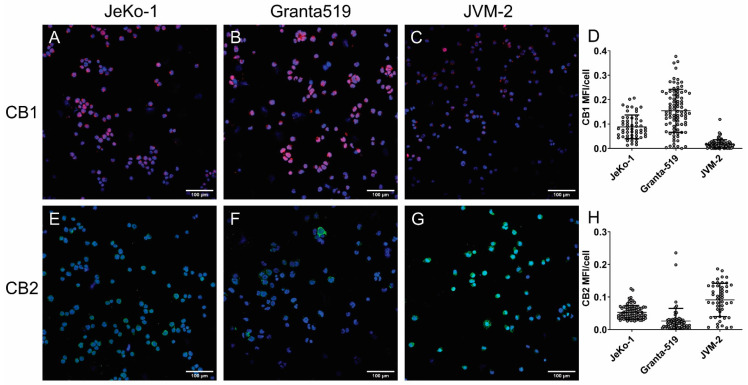
Cannabinoid receptor protein expression. CB1 expression can be visualized in red (TRITC) in (**A**) JeKo-1, (**B**) Granta-519, and (**C**) JVM-2 and CB2 in the green FITC channel in (**E**) JeKo-1, (**F**) Granta-519 and (**G**) JVM-2. DAPI (blue) is staining the nuclei. Mean fluorescence intensity (MFI) was quantified for each cell (grey circles) and is shown in panels (**D**) for CB1 and (**H**) for CB2.

We aimed at investigating the chemotaxis towards 2-AG and the effect on CXCL12-mediated chemotaxis in JeKo-1 and two additional MCL cell lines. JeKo-1, Granta519 and JVM-2 were chosen due to their establishment from peripheral blood. They all expressed CXCR4 at the cell surface (Supplementary Figure S1C), and as previously described [35,36], they all expressed *CNR2* at the mRNA level, with a higher expression in Granta519 cells (Figure 1B). Of the three cell lines, JVM-2 had the lowest *CNR1* expression, at the limit of the detection level. These cell linese also expressed the cannabinoid receptors CB1 (Figure 4A–C) and CB2 (Figure 4E–G) at the protein level, with the lowest level of CB1 and the highest level of CB2 in JVM-2 (Figure 4D,H, Supplementary Figure S6A,D). 

**Figure 5:**
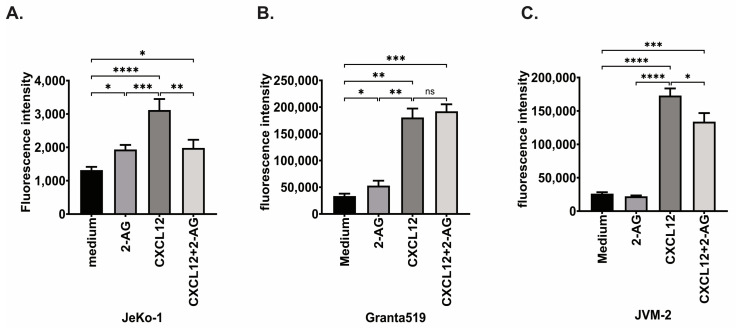
Chemotaxis of MCL cell lines. (**A**) JeKo-1, (**B**) Granta519, (**C**) JVM-2, cells were subjected to chemotaxis towards vehicle or 2-AG (100 nM), CXCL12 (200 ng/mL) or the combination of 2-AG and CXCL12; Paired *t*-test, ns: non-significant, * *p* < 0.05, ** *p* < 0.01, *** *p* < 0.001, **** *p* < 0.0001.

MCL cell lines were then subjected to chemotaxis towards 2-AG +/− CXCL12 (Figure 5A–C). In Figure 2A, we showed that 2-AG induced chemotaxis in JeKo-1, but to a lower level than towards CXCL12. We confirmed this data with the fluorescence intensity read out, in which chemotaxis towards CXCL12 was increased by 2.3-fold compared to vehicle control (*p* = 0.001, Figure 5A). Neither CB1 nor CB2 antagonists affected the chemotaxis towards CXCL12 in JeKo-1 (Supplementary Figure S3). When Granta519 cells were subjected to chemotaxis towards 2-AG, it showed a 1.5-fold increase of chemotaxis of (*p* = 0.03, Figure 5B), while JVM-2 did not migrate more towards 2-AG than towards medium alone (*p* = 0.2, Figure 5C). CXCL12 also induced chemotaxis in both Granta519 and JVM-2, by 5.4-fold (*p* = 0.002) and 4.6-fold (*p* = 0.03), respectively.

When 2-AG was combined with CXCL12, chemotaxis of JeKo-1 and JVM-2 cells was significantly reduced compared with CXCL12 alone by 1.6-fold for JeKo-1 (*p* < 0.05) and 1.3-fold for JVM-2 (*p* = 0.007). This reduction of the CXCL12-mediated chemotaxis by 2-AG was similar to the majority of MCL primary cells. However, the chemotaxis of Granta519 towards the combination of 2-AG and CXCL12 was not significantly different from chemotaxis towards CXCL12 alone (*p* = 0.13), which was also the case for some of the primary samples (MCL and CLL) (Figure S4).

### 3.6. Effect of 2-AG on CXCR4 Surface Expression

One possible explanation of how 2-AG could modulate the chemotaxis to CXCL12 is to impair the ability of the CXCR4 receptor to internalize and signal upon CXCL12 binding, as described in breast and prostate cancer [37]. To investigate this possibility, we quantified CXCR4 membrane expression in MCL and CLL samples by flow cytometry analysis. There was no difference in the basal CXCR4 expression (median fluorescence intensity, MFI) between MCL and CLL (Supplementary Figure S5A). All samples expressed functional CXCR4 at the cell surface, as shown by the expression being reduced by 30 +/− 20% after 10 min incubation with CXCL12 (Supplementary Figure S5B), concordant with previous reports [37,38,39,40]. CXCR4 surface expression was also measured after 10 min incubation with 2-AG, and we concluded that 2-AG did not affect the expression of CXCR4 compared with incubation with medium alone in either disease type (*p* = 0.11 for MCL and *p* = 0.77 for CLL). Furthermore, 2-AG + CXCL12 did not significantly change CXCR4 expression as compared with CXCL12 alone (*p* = 0.41 for MCL and *p* = 0.47 for CLL; Supplementary Figure S5B).

**Figure 6:**
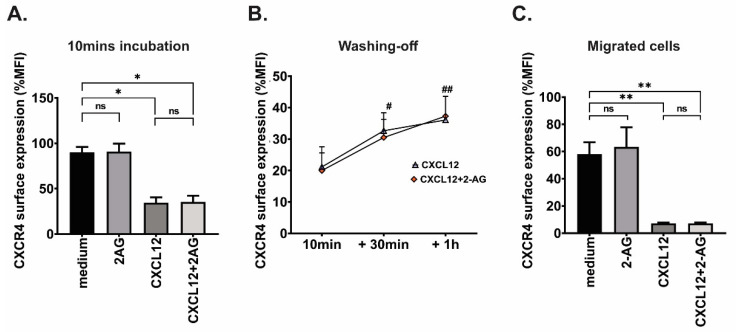
CXCR4 surface expression in JeKo-1 cells (**A**) after 10 min incubation with 2-AG (100 nM), CXCL12 (200 ng/mL) or the combination of 2-AG and CXCL12 and (**B**) after washing off cells, then re-incubation for 30 min and 1 h with medium alone, shown as percentage of MFI normalized to non-treated cells. Paired *t*-test, ns: non-significant, * *p* < 0.05, Unpaired *t*-test, # *p* < 0.05 between 10 min and +30 min, ## *p* < 0.01 between 10 min and +1 h. (**C**) CXCR4 surface expression JeKo-1 cells after being subjected to 4 h chemotaxis towards the same conditions, shown as percentage of MFI normalized to non-migrated cells; Paired *t*-test, ns: non-significant, * *p* < 0.05, ** *p* < 0.01.

In addition, we measured the CXCR4 surface expression in JeKo-1 cell line after 10 min incubation (Figure 6A). We did not observe any difference in the CXCR4 expression at the cell surface upon pre-incubation with CXCL12 alone or in combination with 2-AG. We also investigated the re-expression of the receptor after CXCL12 treatment (recycling of CXCR4) in JeKo-1 cell line (Figure 6B). This was done by first incubating the cells with CXCL12 for 10 min, then washing the cells and measuring the CXCR4 expression at 30 min and 1 h after incubating the cells in medium alone. We could first confirm that the CXCR4 expression increased after washing off, and that this re-expression was not significantly different in cells exposed to the combination of CXCL12 + 2-AG. Lastly, we verified that the CXCR4 at the cell surface was not differentially expressed in migrated cells harvested after 4 h chemotaxis (Figure 6C). Altogether, the data suggests that the inhibition of CXCL12-mediated chemotaxis by 2-AG is not due to an impaired internalization or re-expression of the CXCR4 receptor.

### 3.7. 2-AG and CXCL12 Combination Impact on the p38, Akt and p44/42 Signaling Pathways

**Figure 7:**
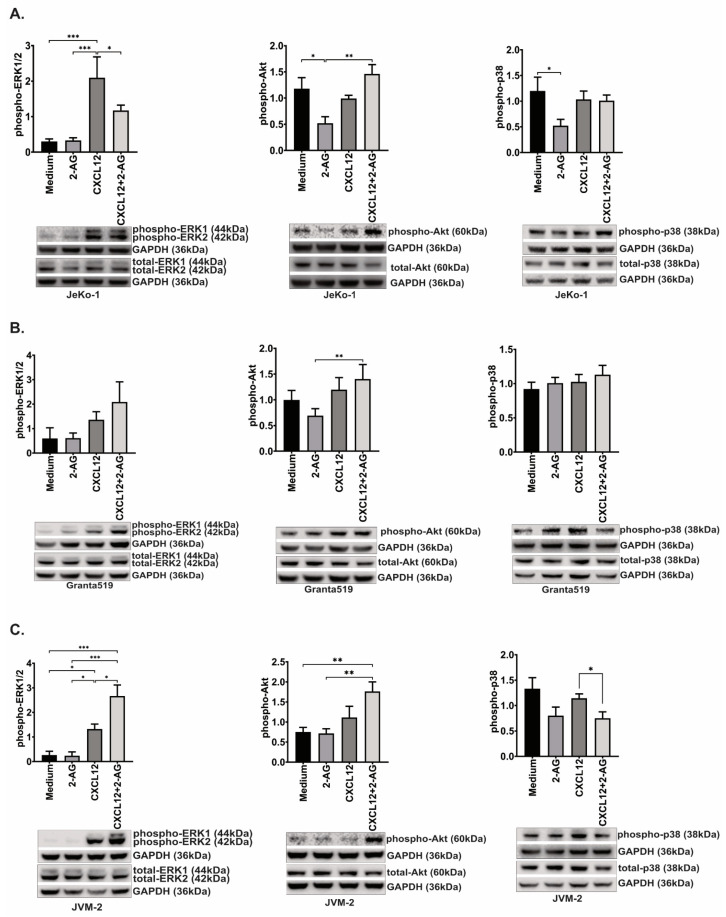
Activation of signaling pathways. (**A**) JeKo-1, (**B**) Granta519 and (**C**) JVM-2 were incubated for 2 min with CXCL12 (200 ng/mL) or 2-AG (100 nM) or the combination, and the activation of the signaling pathways ERK1/2, Akt and p38 was assessed by Western blotting, normalizing phospho/GAPDH band intensity to total/GAPDH as described in Section 2, bars represent an average of the ratio of at least four repeats and error bars are standard error of the mean; paired *t*-test, * *p* < 0.05, ** *p* < 0.01, *** *p* < 0.001, ns: non-significant, a representative blot for each experiment is shown. Original blot see Supplementary File S1.

In order to investigate potential crosstalk between CB1, CB2 andCXCR4 we focused on pathways previously described to mediate signals from CXCR4 to induction of chemotaxis in lymphoma cells [1,41], and from the cannabinoid receptors upon stimulation [24,42]. We treated the cells with the 2-AG and CXCL12 separately or in combination for 2 min at 37 °C and investigated the early activation of p38, p44/42 (ERK1/2) and Akt signaling pathways (Figure 7).

After 2 min incubation with CXCL12, we could observe a significant increase in p44/42 phosphorylation in JeKo-1 (7-fold increase, *p* < 0.001; Figure 7A) and JVM-2 (5-fold increase, *p* = 0.03; Figure 7C) but not Granta519 (*p* = 0.3; Figure 7B). 2-AG alone reduced the phosphorylation of p38 and Akt in JeKo-1 (2.3-fold decrease, *p* = 0.03 and *p* = 0.02, respectively).

**Figure 8:**
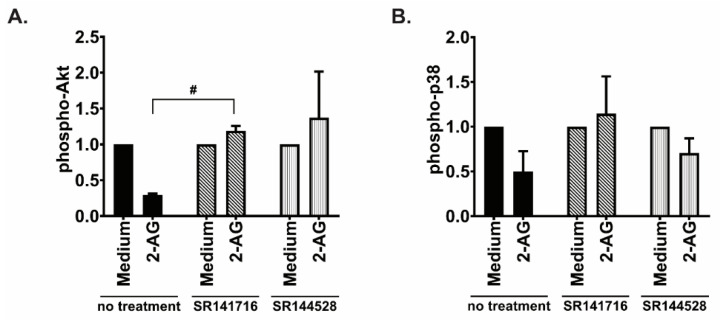
Effects of inhibiting the cannabinoid receptors on Akt and p38 activation. JeKo-1 cells were treated for 20 min with the CB1 antagonist SR141716 (10 nM), or with the CB2 inverse agonist SR1445282 (10 nM), prior to be incubated for 2 min with 2-AG. Activation of the signaling pathways (**A**) Akt and (**B**) p38 was assessed by Western blotting, normalizing phospho/GAPDH band intensity to total/GAPDH as described in Section 2, bars represent an average of the ratio of three repeats and error bars are standard error of the mean; 2-AG condition is shown as normalized to incubation with vehicle set as 1, unpaired *t*-test with Welch’s correction, # *p* < 0.05.

Inhibition of the phosphorylation of Akt was reversed after CB1 antagonist SR141716 and CB2 inverse agonist SR144528 (Figure 8A), while only CB1 antagonist SR141716 restored fully p38 phosphorylation (Figure 8B). 2-AG did not change phosphorylation status in Granta519, although it induced 1.5-fold increased chemotaxis compared to medium alone (Figure 5B). 2-AG did not significantly change the phosphorylation status of these three investigated pathways in JVM-2 (Figure 7C), concordant with the chemotaxis data that was unchanged towards 2-AG alone in this cell line (Figure 5C).

The combination of 2-AG and CXCL12 had a contrasting effect on the phosphorylation status of ERK1/2, in JeKo-1 and JVM-2. In JeKo-1, ERK1/2 phosphorylation significantly changed after incubation with the two compounds together (1.8-fold decrease, *p* = 0.04, Figure 7A), while in JVM-2, 2-AG combined with CXCL12 significantly increased the phosphorylation status of ERK1/2 (2-fold increase, *p* = 0.01; Figure 7C) and decreased p38 phosphorylation (1.4-fold decrease, *p* = 0.04) compared with CXCL12 alone. The combination of CXCL12 and 2-AG seemed to increase the phosphorylation of Akt pathway in Jeko-1 and JVM-2; however, this was not statistically significant. The activation of the signaling pathways in Granta519 were fluctuating and did not show any significant differences between the different conditions.

**Figure 9:**
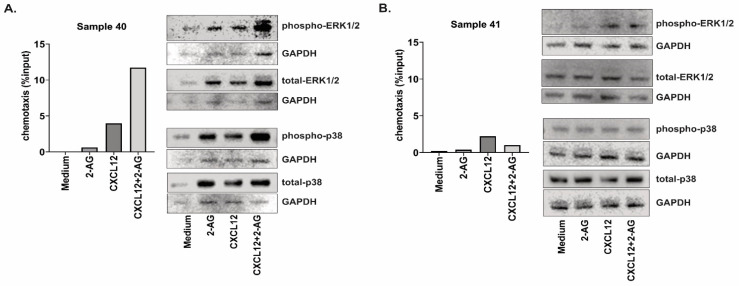
Signaling pathways activation in primary CLL samples. CLL cells from (**A**) Sample 40 and (**B**) Sample 41 were incubated for 2 min with CXCL12 (200 ng/mL) or 2-AG (100 nM) or the combination, and the activation of the signaling pathways ERK1/2 and p38 was assessed by Western blotting. The bar graphs represent the chemotaxis data for the two samples, towards medium, 2-AG (100 nM), CXCL12 (200 ng/mL) or the combination, shown as % input cells after 4 h chemotaxis. Original blot see Supplementary File S1.

In addition, we could investigate the activation of the pathways ERK1/2 and p38 in two CLL samples that displayed different chemotaxis patterns towards the combination CXCL12 + 2-AG (Figure 9). Both samples displayed an increased chemotaxis towards 2-AG and towards CXCL12 compared with medium alone. However, sample 40 displayed an increased chemotaxis towards the combination of 2-AG and CXCL12 compared to CXCL12 alone (3-fold increase), while sample 41 had a decreased chemotaxis towards the combination (2-fold decrease). Both samples expressed *CNR1* and *CNR2* (Figure 1C,D), with sample 40 expressing *CNR1* at lower levels than B cells from buffy coat and sample 41 expressing *CNR1* at a 2.8-fold increase compared to buffy coat B cells. The activation of the signaling pathways seems to be affected by the combination of 2-AG and CXCL12 in the case of Sample 40, but not in sample 41.

## 4. Discussion

In this study we demonstrate that 2-AG induces chemotaxis of MCL and CLL primary cells isolated from blood, and in the MCL cell lines JeKo-1 and Granta519, however, to a lesser extent than CXCL12. Other reports also showed that 2-AG induces chemotaxis in different cell types, such as NK cells [43], monocytes [44] and eosinophils [45] from peripheral blood, as well as CD34+ hematopoietic stem cells [27] and mesenchymal stromal cells [46], from bone marrow. 2-AG was also reported to increase the motility of the T cell line Jurkat in a co-culture system with stromal cells pre-incubated with 2-AG [47]. In mice, 2-AG has a role in the innate immune response by increasing the recruitment of dendritic cells to the lymph node upon immunization [48] and has a different chemotactic potency according to the activation state of B lymphocytes [49]. Importantly, the cells used in these previous reports express mainly CB2 receptor but none or low levels of CB1. Our study is the first to scrutinize this in primary lymphoma cells, which express both cannabinoid receptor types at higher levels than normal B lymphocytes. Both CB1 and CB2, demonstrated to be expressed at the protein level in MCL cell lines, are involved in the chemotaxis towards 2-AG as shown by using specific CB1 and CB2 inhibitors (Figure 1). In our hands, silencing the genes was not a useful tool as downregulation of *CNR2* induced a change in *CNR1* gene expression, which suggests a crosstalk between the regulation of these two genes. We therefore chose to use specific antagonists to CB1 and CB2 which demonstrated that both receptors are involved in 2AG induced chemotaxis. We also show that the selective CB2 inverse agonist SR144528 increases the basal chemotaxis of B lymphocytes. The fact that blocking the CB2 receptor induces spontaneous chemotaxis is concordant with previous reports that show the importance of CB2 in the retention of the cells in lymphoid compartments [20,21,22].

Endocannabinoids, chemokines and adhesion molecules participate in the communication between the tumor cells and their microenvironment. Each of these have specific functions. CXCR4 and CXCR5 are implicated in the homing of cells into lymphoid tissues where their respective ligands CXCL12 and CXCL13 are found in high concentrations [50,51]. Within the tissue, adhesion molecules are involved in the engraftment and migration of the tumor cells beneath the stromal cells [1], enhancing the risk of drug resistance and relapses. Endocannabinoids are released in the bone marrow microenvironment by mesenchymal cells [27], including stromal cells and osteoblasts, which might influence the response of T lymphocytes in that microenvironment [46,51]. In the blood circulation, chemokines are present in lower concentration than in lymphoid tissues, but 2-AG is elevated in the plasma of cancer patients [25,26]. In this context, we investigated whether the combination of 2-AG and CXCL12 would have a synergistic effect on chemotaxis, as some cytokines, such as interleukin-5 and interleukin-3, do when combined with 2-AG in hematopoietic cells, B lymphocytes and neutrophils [52,53,54]. We show here that the combination of 2-AG and CXCL12 had a different effect on chemotaxis in CLL than in MCL as it reduced chemotaxis in most MCL cases and increased it in CLL. A previous report from Coopman et al. has also investigated the combination of CXCL12 and 2-AG. Although their experimental set-up was different from ours, as they used T lymphocytes that were incubated with 2-AG prior to subjecting them to chemotaxis towards CXCL12, a similar conclusion as for MCL cells from this study was reached, although T cells do not express CB1 receptor [55].

CB2 has been described to heterodimerize with the CXCR4 receptor in breast cancer and prostate cancer cells when both receptors are simultaneously activated by respective agonists. This heterodimerization blocks CXCL12-induced CXCR4 internalization and downstream signaling [37]. Our results suggest different mechanisms behind the effects of 2-AG on CXCL12-induced migration in lymphoma cells. In contrast to findings described in breast cancer, in MCL and CLL cells CXCR4 was still internalized even when CXCR4, CB1 and CB2 were stimulated simultaneously by their endogenous ligands. The fact that the CXCR4 receptor could still internalize upon activation and that the recycling of CXCR4 was not affected by incubation with 2-AG or antagonists to CB1 or CB2, indicates that the reduced migration is rather caused by alteration in the downstream signaling or by the activation of alternative pathways than by affecting CXCR4 receptor functionality and localization.

In our hands, 2-AG did not induce activation of ERK1/2 pathway in lymphoma cells, in contrast to reports on other cell types such as CB2-transfected CHO cells and CB1 and CB2 expressing microglial cells [47,56,57]. This could be due to different cell types studied and/or due to different concentrations of 2-AG used in our study and studies by others. We used 100 nM, which is comparable to the physiological concentration in blood and tissues [25,27], similarly used in a study by Wang et al., in which low 2-AG concentrations in astrocytes do not induce ERK1/2 activation [58] but induced chemotaxis. In fact, it seems that ERK1/2 activation requires micromolar (µM) of 2-AG, as shown in T lymphocytes [55]. However, Akt phosphorylation was found to be inhibited with the presence of 2-AG, which was reverted with CB1 and CB2 inhibitors. Similar results have been described in B-acute lymphoblastic leukemia cells after CB2 stimulation with the specific agonist JWH-133 [59]. The effects of 2-AG on CXCL12-mediated chemotaxis and on the downstream signaling pathways are different in the three MCL cell lines used in this study, reflecting the variation in chemotaxis in primary samples. However, it seems that for JeKo-1 and JVM-2, 2-AG is affecting CXCL12-mediated ERK1/2 phosphorylation, which could explain the inhibitory effect on the chemotaxis (Figure 4B,C). The fact that JeKo-1 and JVM-2 had a different pattern on the ERK1/2 and p38 signaling after the combination of 2-AG and CXCL12 could be due to the different expression of the cannabinoid receptors, as suggested by Kleyer et al. and Callén et al. [60,61]. *CNR1* mRNA and its protein expression are very low/negative in JVM-2, and we see less effect on the chemotaxis towards the combination of CXCL12 and 2-AG in that cell line compared with JeKo-1, which has higher *CNR1* mRNA and protein levels. We hypothesize that both receptors are implicated in the chemotaxis response to the combination of CXCL12 and 2AG. This could be one explanation for the difference in 2-AG modulation of CXCL12 response between JeKo-1 and JVM-2 regarding pERK signaling. We could then further hypothesize that the differences in clinical presentation between MCL (being mainly restricted to lymph nodes and bone marrow) and CLL (leukemic disease) could in part be due to their differential expression of *CNR1* and *CNR2*.

## 5. Conclusions

Here, we describe a novel player in lymphoma biology. 2-AG on its own is inducing the chemotaxis of most lymphoma cells and it is modulating the CXCL12-mediated chemotaxis that, based on our findings, is dependent on CB1 and CB2 expression levels. These novel findings in hematological malignancies strengthen the potential role of the endocannabinoid system as playing an important role in the positioning of lymphoma cells in tissues and their release in the blood circulation. Our results suggest that, together with other chemokines, 2-AG is involved in the regulation of circulating cells in the blood. Further research is, however, still needed to confirm that targeting 2-AG synthesis in lymphoma would be a potential therapeutic strategy.

## Figures and Tables

**Table 1:** Clinical and biological features of MCL and CLL samples included in the study for chemotaxis experimental part.

Sample ID	Age (y)	Sex (F Female; M Male)	Diagnosis	Lymphocyte Count (×10^9^/L)	Other Malignancy/Immune Disease	Treated (1 Yes, 0 No)	Time to Treatment from Diagnosis (Months)
1	72	M	MCL	20	-	0	-
2	64	M	CLL	19.5	-	0	-
3	69	M	CLL	66.4	-	0	-
4	75	F	CLL	42.6	-	0	-
6	48	M	CLL	144.9	-	0	-
10	73	F	CLL	21.3	-	0	-
11	77	M	CLL	27.7	-	0	-
12	56	M	CLL	129.9	-	0	-
13	73	M	CLL	103.8	-	1	1
14	77	F	CLL	66	-	0	-
15	81	F	CLL	22.5	-	1	84
16	47	F	CLL	11	-	0	-
17	69	M	CLL	17.3	-	0	-
18	72	M	MCL	50.5	-	0	-
19	55	M	CLL	54	-	0	-
20	81	M	CLL	93.3	-	1	7
21	80	M	CLL	11.1	1	0	-
22	38	F	CLL	216.6	-	0	-
25	87	M	MCL	60.6	polymyalgia rheumatica	0	-
26	71	M	CLL	10.4	-	0	-
27	59	F	CLL	84	-	0	-
30	78	M	MCL	not known	-	not known	-
31	77	F	CLL	141	-	0	-
32	81	F	MCL	89	-	0	-
40	58	F	CLL	100	-	0	-
41	44	M	CLL	335	-	0	-
42	78	F	CLL	114	-	1	66

## Data Availability

Raw data are available from the corresponding authors upon reasonable request.

## Supplementary Materials

The following are available online at https://www.mdpi.com/article/10.3390/cancers15051585/s1, Figure S1: (A) Gating strategy to quantify the efficiency of RosetteSep to enrich the lymphoma cells. Cells were gated on CD45 positive cells, and CD19+CD5+ cells were quantified. CXCR4 was also quantified with flow cytometry method and median fluorescence intensity (MFI) was used for the quantification. (B) Chemotaxis of primary MCL and CLL cells grouped together towards 2-AG; paired sample Wilcoxon signed rank test, *** *p* < 0.001. (C) CXCR4 surface expression in JeKo-1, Granta519 and JVM-2 analysis by flow cytometry. Figure S2: (A) Standard curve of calcein-AM stained cells and the fluorescence intensity in JeKo-1, Granta519 and JVM-2 cell lines. (B) *CNR1* and *CNR2* mRNA expression after specific siRNA; paired *t*-test, * *p* < 0.05, ** *p* < 0.01, **** *p* < 0.0001, ns: non-significant. (C) Chemotaxis of JeKo-1 cells treated with *CNR1* or *CNR2* siRNA towards medium or 2-AG (100 nM), n = 3. (D) JeKo-1 cell proliferation and viability after electroporation of specific siRNA; paired *t*-test, * *p* < 0.05, ** *p* < 0.01, *** *p* < 0.001, **** *p* < 0.0001. Figure S3: Chemotaxis towards CXCL12 after incubation for 20 min with (A) the CB1 antagonist SR141716 (10 nM) or with (B) the CB2 inverse agonist SR144528 (10 nM), in JeKo-1 cell line. Figure S4: Effect of 2-AG titration on the phospho-pathways. JeKo-1 cells were incubated for 2 min with CXCL12 (200 ng/mL) or 2-AG at three different concentrations, 100 nM, 1 µM and 10 µM, and the activation of the signaling pathways ERK1/2, Akt and p38 was assessed by Western blotting, normalizing phospho/GAPDH band intensity to total/GAPDH as described in Section 2, bars represent an average of the ratio of at least four repeats and error bars are standard error of the mean; paired *t*-test, ** *p* < 0.01, **** *p* < 0.0001; a representative blot for each experiment is shown. Figure S5: (A) Effects of 2-AG on CXCR4 surface expression in primary MCL and CLL cells. Basal CXCR4 surface expression in MCL and CLL cells are shown as median fluorescence intensity (MFI); Mann–Whitney test, ns: non-significant. (B) CXCR4 surface expression after 10 min incubation with 2-AG (100 nM), CXCL12 (200 ng/mL) or the combination of 2-AG and CXCL12 in MCL and CLL samples, shown as normalized to incubation with vehicle set as 1; Paired *t*-test, ns: non-significant, ** *p* < 0.01, *** *p* < 0.001, **** *p* < 0.00001. Figure S6: Cannabinoid receptor expression. (A) CB1 and (D) CB2 protein expression in the MCL cell lines JVM-2 and JeKo-1, in the T cell line Jurkat and in cortex protein lysate (Novus Biologicals), by Western blotting, as well as in (B) the brain cell line U-251 and (E) Jurkat by immunofluorescence. Respective mRNA expression is shown in (C) *CNR1* and (F) *CNR2*. Table S1: Patients’ characteristics and experiments performed on each sample (chemotaxis, CXCR4 expression). File S1: Original blot.
