# Supplementary material for: 2-Arachidonoylglycerol Modulates CXCL12-Mediated Chemotaxis in Mantle Cell Lymphoma and Chronic Lymphocytic Leukemia
Source: Cancers (Basel). 2023 Mar 3;15(5):1585. doi: 10.3390/cancers15051585 (PMC10000973; doi:10.3390/cancers15051585)

Supplementary Table and Figures for manuscript Merrien et al., 2-arachidonoylglycerol modulated the CXCL12-mediated chemotaxis in mantle cell lymphoma and chronic lymphocytic leukemia.

Supplementary Table S1. Patients' characteristics and experiments performed on each sample (chemotaxis, CXCR4 expression).

Patient ID	Diagnosis	Lymphocyte count (x10^9/L)	Proportion of tumour cells (CD45+CD19+CD5+) (%)	Chemotaxis data (y yes, n no)	CXCR4 surface expression after treatments data (y yes, n no)
1	MCL	20	98,1	y	n
2	CLL	19,5	98,5	y	n
3	CLL	66,4	87,2	y	n
4	CLL	42,6	98,9	y	n
5	MCL	5,9	27*	n	y
6	CLL	144,9	95,3	y	y
7	MCL	58	78,7**	n	y
8	CLL	25,2	97,1**	n	y
9	CLL	55,8	98,3**	n	y
10	CLL	21,3	96,5	y	y
11	CLL	27,7	99,8	y	y
12	CLL	129,9	95,1	y	n
13	CLL	103,8	98,6	y	n
14	CLL	66	94,1	y	y
15	CLL	22,5	94,2	y	n
16	CLL	11	95,7	y	n
17	CLL	17,3	93,5	y	n
18	MCL	50,5	99,1	y	n
19	CLL	54	97,7	y	n
20	CLL	93,3	98,7	y	n
21	CLL	11,1	97,4	y	n
22	CLL	216,6	99,6	y	n
23	blastoid MCL	20	92,5	n	y
24	MCL	27	95,8	n	y
25	MCL	60,6	98,1	y	y
26	CLL	10,4	86,9	y	n
27	CLL	84	99,5	y	n
28	CLL	27	93,5	n	y
29	CLL	18	98,4	n	y
30	MCL	not known	99,6	y	y
31	CLL	141	99,7	y	y
32	MCL	89	99,3	y	y
33	CLL	41,2	81,4**	n	y
34	CLL	17,9	68,4**	n	y
35	CLL	41,4	95,7**	n	y
36	CLL	6,9	97**	n	y
40	CLL	100	99.3	y	n
41	CLL	335	97.5	y	n
42	CLL	114	99	y	n

Supplementary Table and Figures for manuscript Merrien et al., 2-arachidonoylglycerol modulated the CXCL12-mediated chemotaxis in mantle cell lymphoma and chronic lymphocytic leukemia.

Supplementary Figure S1: A) Gating strategy to quantify the efficiency of RosetteSep to enrich the lymphoma cells. Cells were gated on CD45 positive cells, and CD19+CD5+ cells were quantified. CXCR4 was also quantified with flow cytometry method and median fluorescence intensity (MFI) was used for the quantification. **B)** Chemotaxis of primary MCL and CLL cells grouped together towards 2-AG; paired sample Wilcoxon signed rank test, ***p<0.001. **C)** CXCR4 surface expression in JeKo-1, Granta519 and JVM-2 analysis by flow cytometry.

Supplementary Figure S2: A) Standard curve of calcein-AM stained cells and the fluorescence intensity in JeKo-1, Granta519 and JVM-2 cell lines. **B)** *CNR1* and *CNR2* mRNA expression after specific siRNA; paired *t*-test, *p<0.05, **p<0.01, ****p<0.0001, ns: non-significant. **C)** Chemotaxis of JeKo-1 cells treated with *CNR1* or *CNR2* siRNA towards medium or 2-AG (100nM), n=3. **D)** JeKo-1 cell proliferation and viability after electroporation of specific siRNA; paired *t*-test, *p<0.05, **p<0.01, ***p<0.001, ****p<0.0001.

Supplementary Figure S3: Chemotaxis towards CXCL12 after incubation for 20 minutes with A) the CB1 antagonist SR141716 (10nM) or with B) the CB2 inverse agonist SR144528 (10nM), in JeKo-1 cell line.

Supplementary Figure S4: Effect of 2-AG titration on the phospho-pathways. JeKo-1 cells were incubated for 2 minutes with CXCL12 (200ng/ml) or 2-AG at three different concentrations: 100nM, 1µM and 10µM, and the activation of the signaling pathways ERK1/2, Akt and p38 was assessed by Western blotting, normalizing phospho/GAPDH band intensity to total/GAPDH as described in the material and methods, bars represent an average of the ratio of at least four repeats and error bars are standard error of the mean; paired t-test, **p<0.01, ****p<0.0001; a representative blot for each experiment is shown.

Supplementary Figure S5: A) Effects of 2-AG on CXCR4 surface expression in primary MCL and CLL cells. Basal CXCR4 surface expression in MCL and CLL cells are shown as median fluorescence intensity (MFI); Mann-Whitney test, ns: non-significant. B) CXCR4 surface expression after 10 minutes incubation with 2-AG (100nM), CXCL12 (200ng/ml) or the combination of 2-AG and CXCL12 in MCL and CLL samples, shown as normalized to incubation with vehicle set as 1; Paired t-test, ns: non-significant, **p<0.01, ***p<0.001, ****p<0.00001.

Supplementary Table and Figures for manuscript Merrien et al., 2-arachidonoylglycerol modulated the CXCL12-mediated chemotaxis in mantle cell lymphoma and chronic lymphocytic leukemia.

Supplementary Figure S6: Cannabinoid receptor expression. A) CB1 and D) CB2 protein expression in the MCL cell lines JVM-2 and JeKo-1, in the T cell line Jurkat and in cortex protein lysate (Novus Biologicals), by western blotting, as well as in B) the brain cell line U-251 and E) Jurkat by immunofluorescence. Respective mRNA expression is shown in C) *CNR1* and F) *CNR2*.

Supplementary Table and Figures for manuscript Merrien et al., 2-arachidonoylglycerol modulated the CXCL12-mediated chemotaxis in mantle cell lymphoma and chronic lymphocytic leukemia.

Supplementary Figure S1

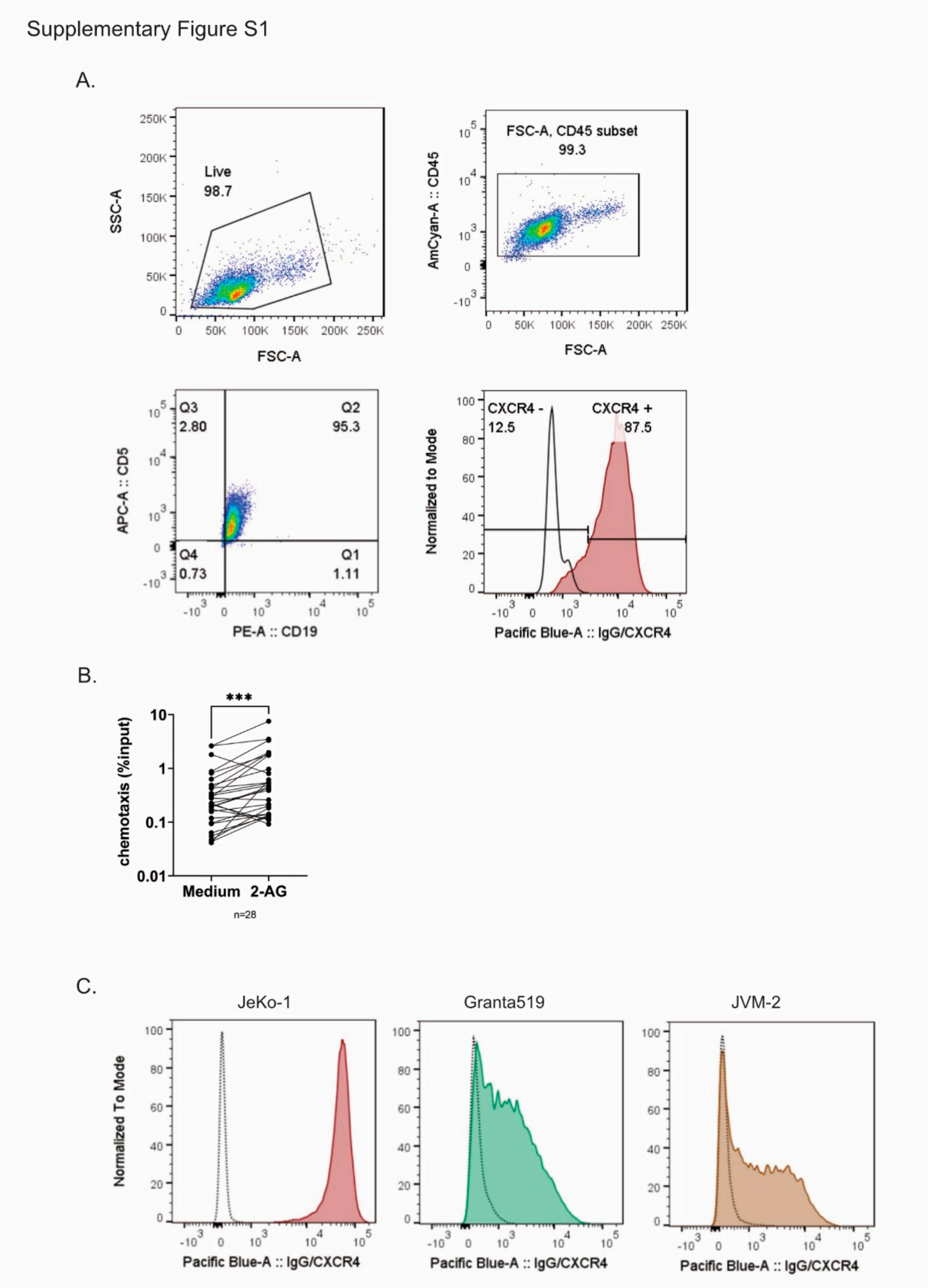

Supplementary Table and Figures for manuscript Merrien et al., 2-arachidonoylglycerol modulated the CXCL12-mediated chemotaxis in mantle cell lymphoma and chronic lymphocytic leukemia.

Supplementary Figure S2

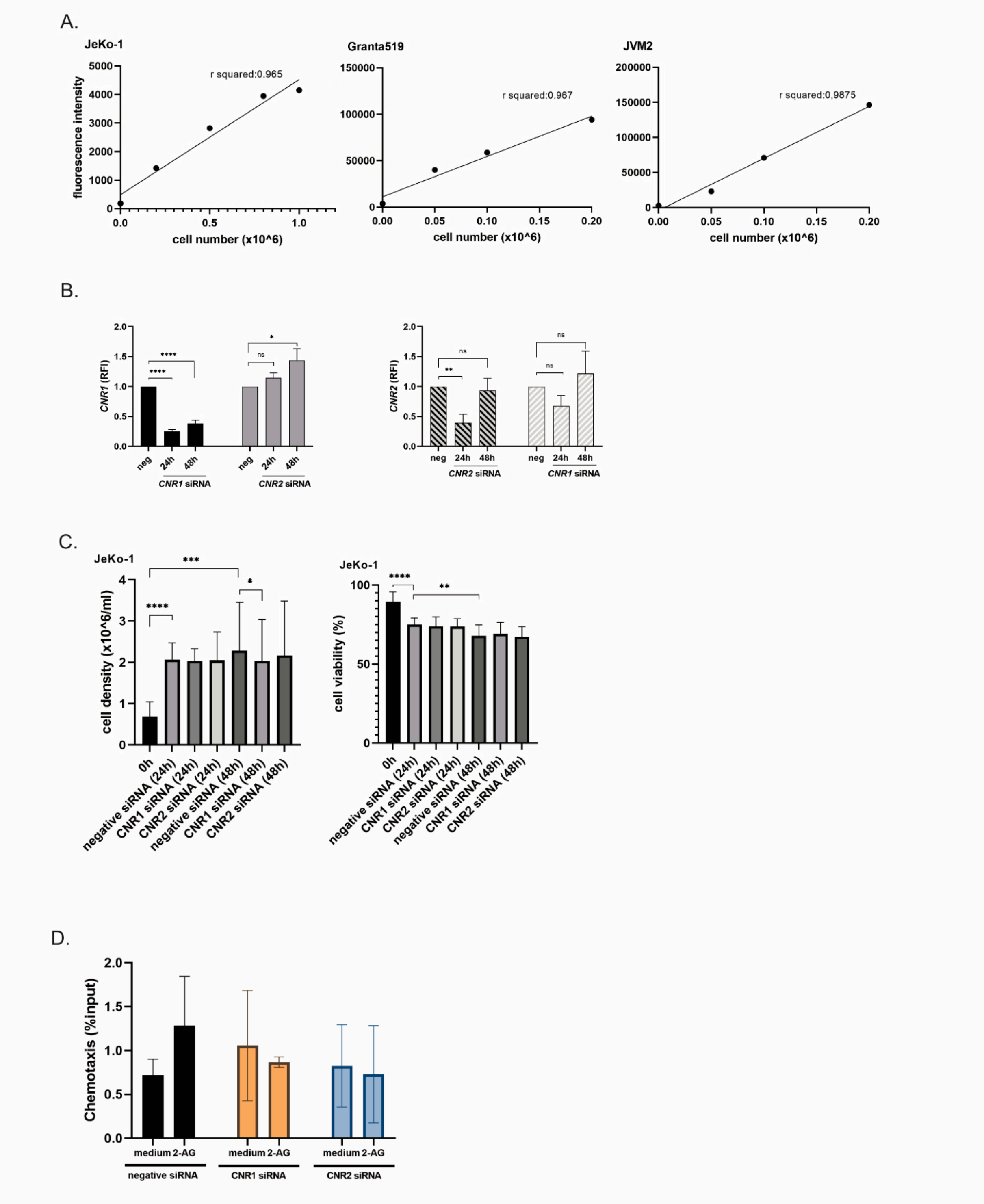

Supplementary Table and Figures for manuscript Merrien et al., 2-arachidonoylglycerol modulated the CXCL12-mediated chemotaxis in mantle cell lymphoma and chronic lymphocytic leukemia.

Supplementary Figure S3

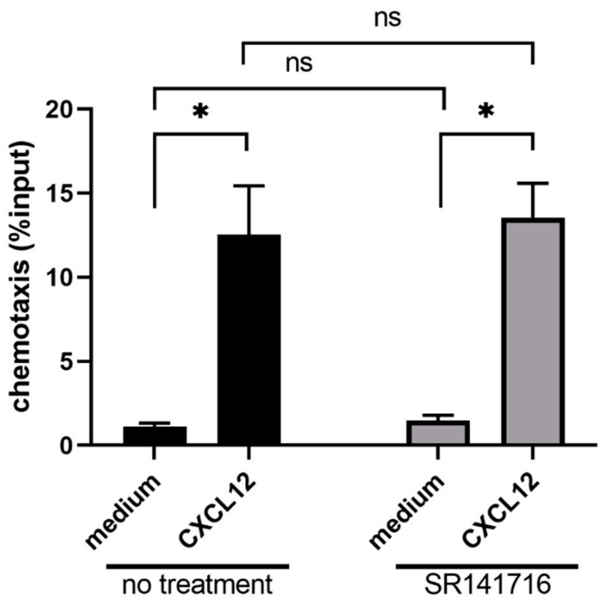

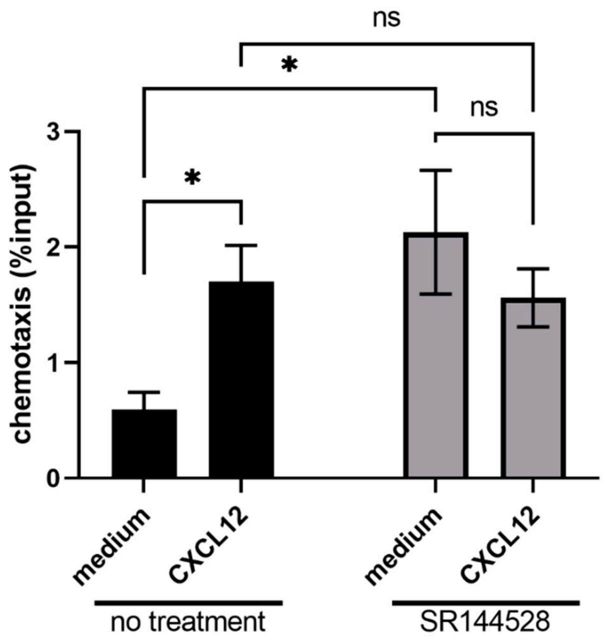

Supplementary Table and Figures for manuscript Merrien et al., 2-arachidonoylglycerol modulated the CXCL12-mediated chemotaxis in mantle cell lymphoma and chronic lymphocytic leukemia.

Supplementary Figure S4

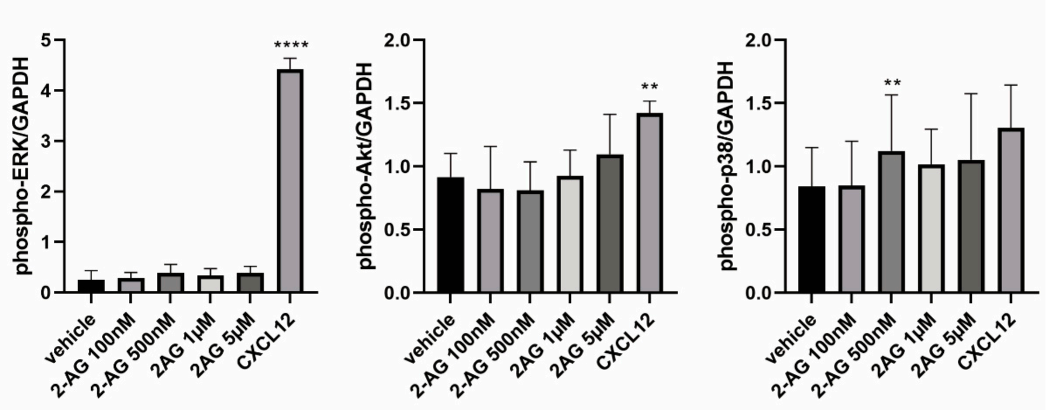

Supplementary Figure S5

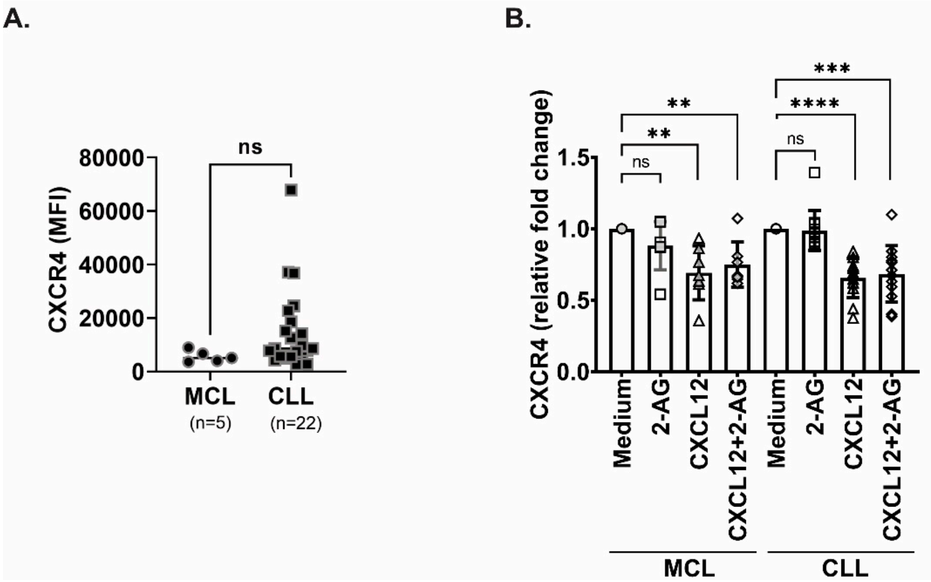

Supplementary Table and Figures for manuscript Merrien et al., 2-arachidonoylglycerol modulated the CXCL12-mediated chemotaxis in mantle cell lymphoma and chronic lymphocytic leukemia.

Supplementary Figure S6

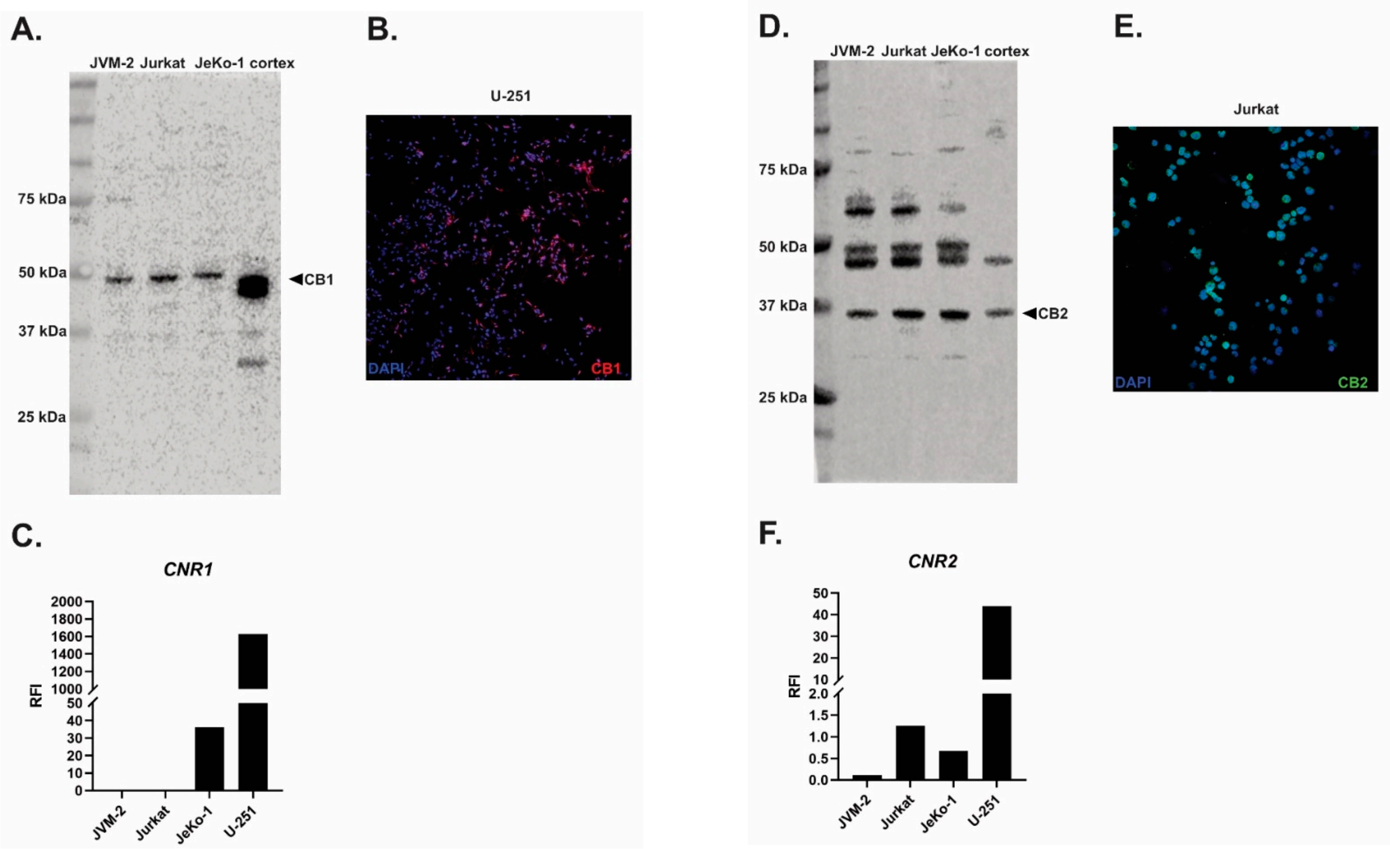

Supplement: Supplementary file 1 [file cancers-15-01585-s001.zip › cancers-2158822-supplementary.pdf]
