# Supplementary material for: 2-Arachidonoylglycerol Modulates CXCL12-Mediated Chemotaxis in Mantle Cell Lymphoma and Chronic Lymphocytic Leukemia
Source: Cancers (Basel). 2023 Mar 3;15(5):1585. doi: 10.3390/cancers15051585 (PMC10000973; doi:10.3390/cancers15051585)
Supplement: Supplementary file 1 [file cancers-15-01585-s001.zip › File S1-Original blot.pdf]

Merrien et al.,
2-arachidonoylglycerol modulates CXCL12-mediated chemotaxis in mantle cell lymphoma and chronic lymphocytic leukemia

Original Images for Blots

Figure 7

JVM-2 cell line

JVM2 – phospho-ERK - 20210414

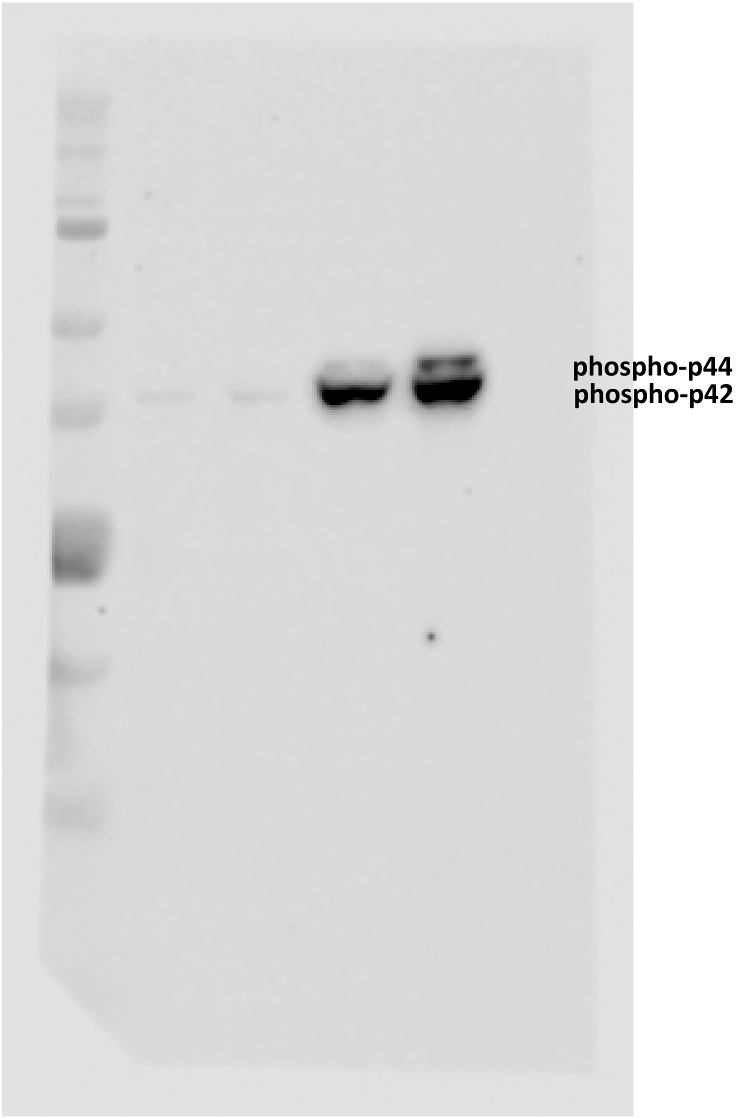

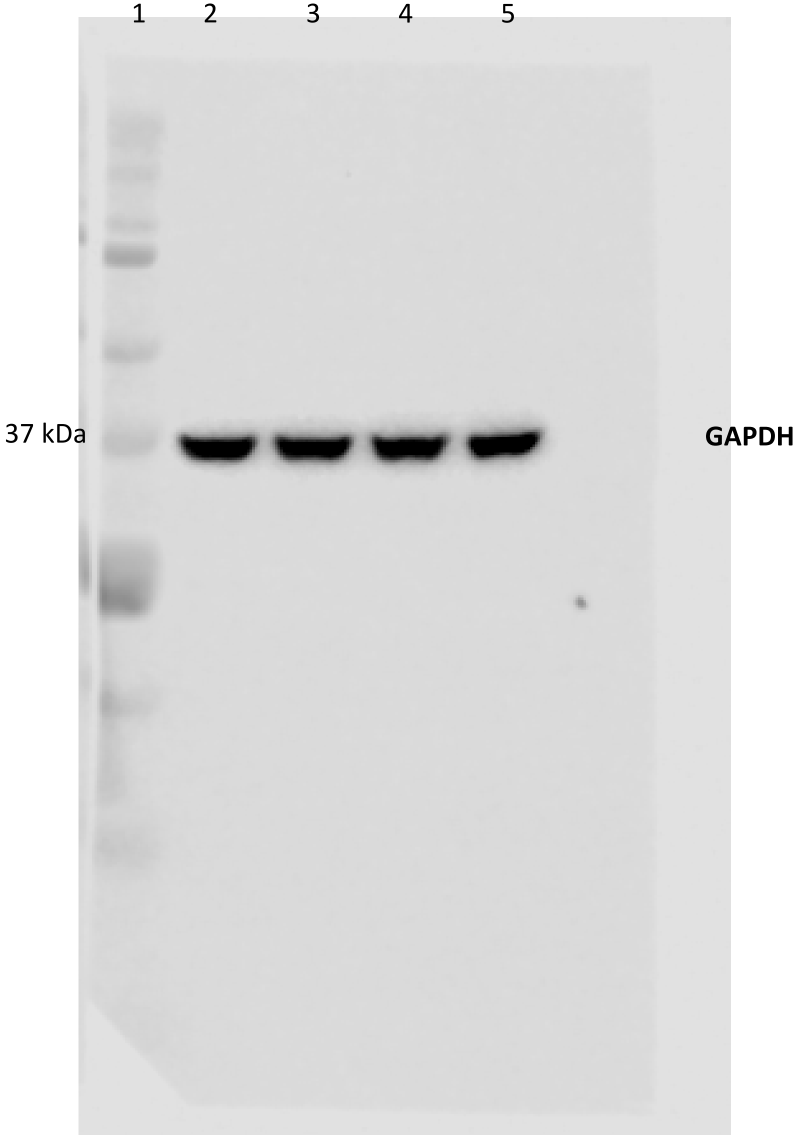

1 ladder

2 vehicle

3 2-AG

4 CXCL12

5 CXCL12+2-AG

JVM-2 cell line

JVM2 – Total-ERK - 20210414

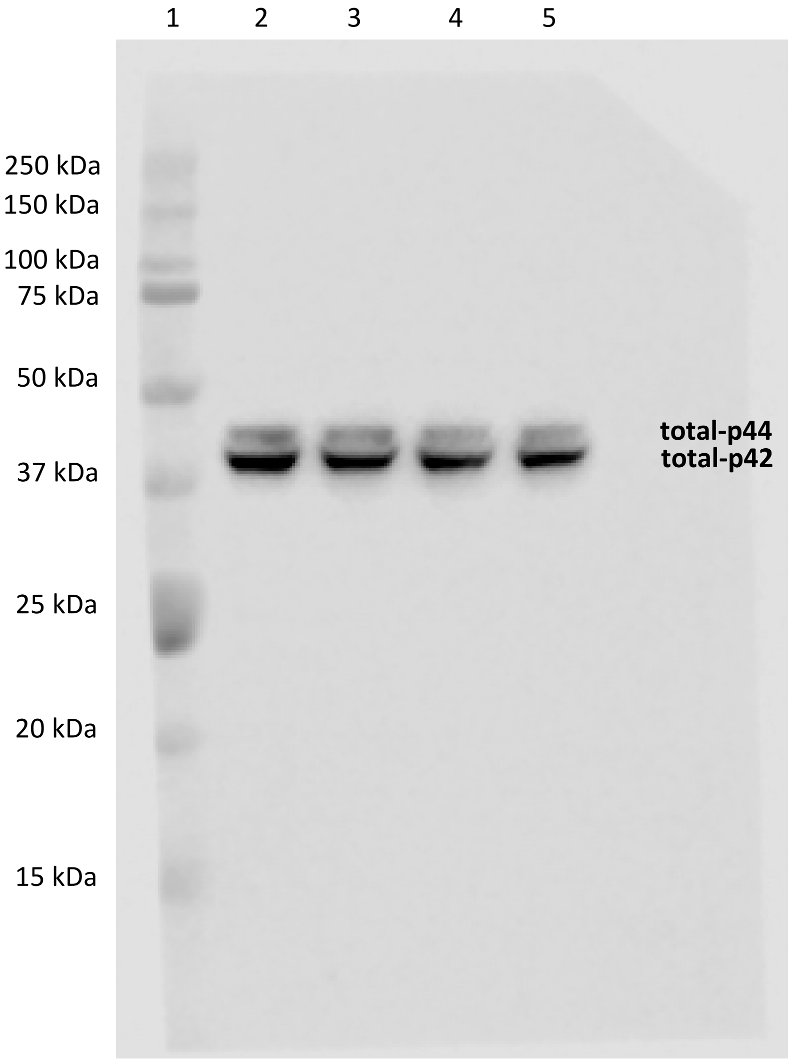

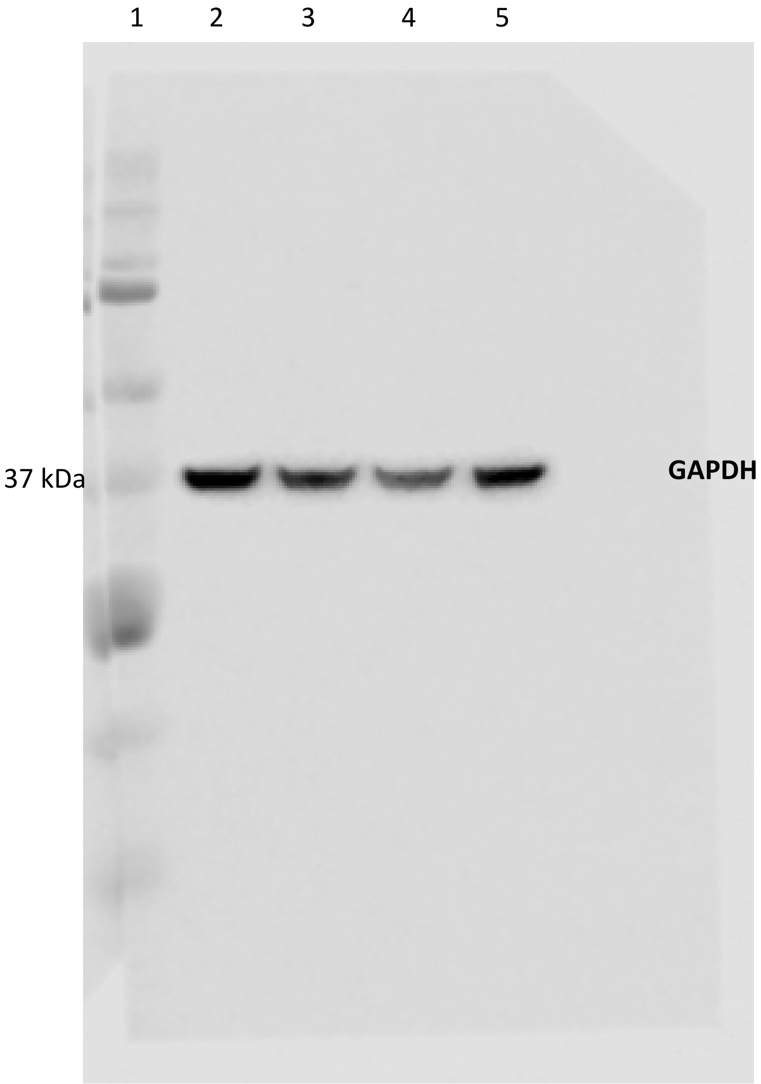

1 ladder

2 vehicle

3 2-AG

4 CXCL12

5 CXCL12+2-AG

JVM-2 cell line

JVM2 – phospho-Akt – 20210414

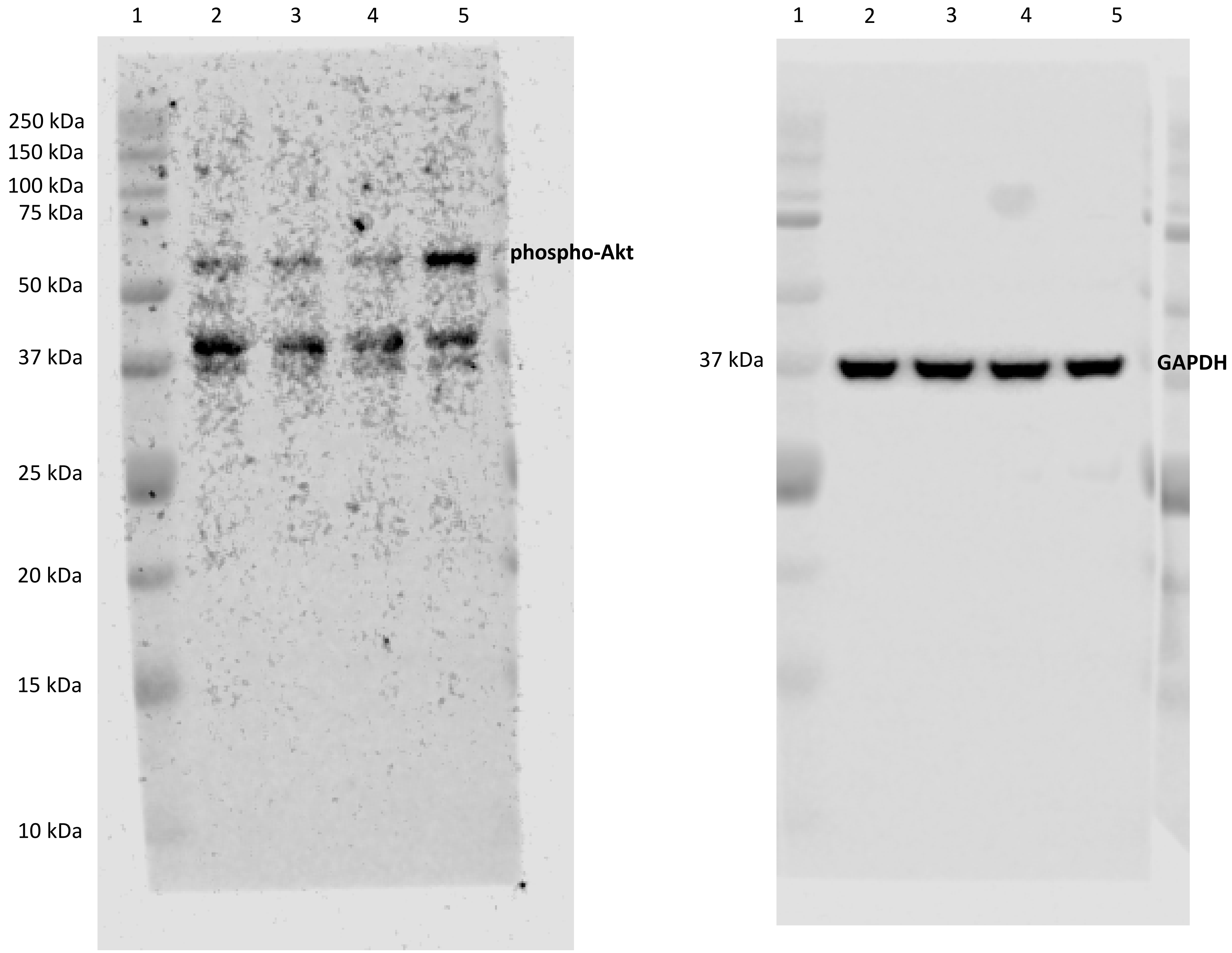

1 ladder

2 vehicle

3 2-AG

4 CXCL12

5 CXCL12+2-AG

JVM-2 cell line

JVM2 – Total-Akt – 20210414

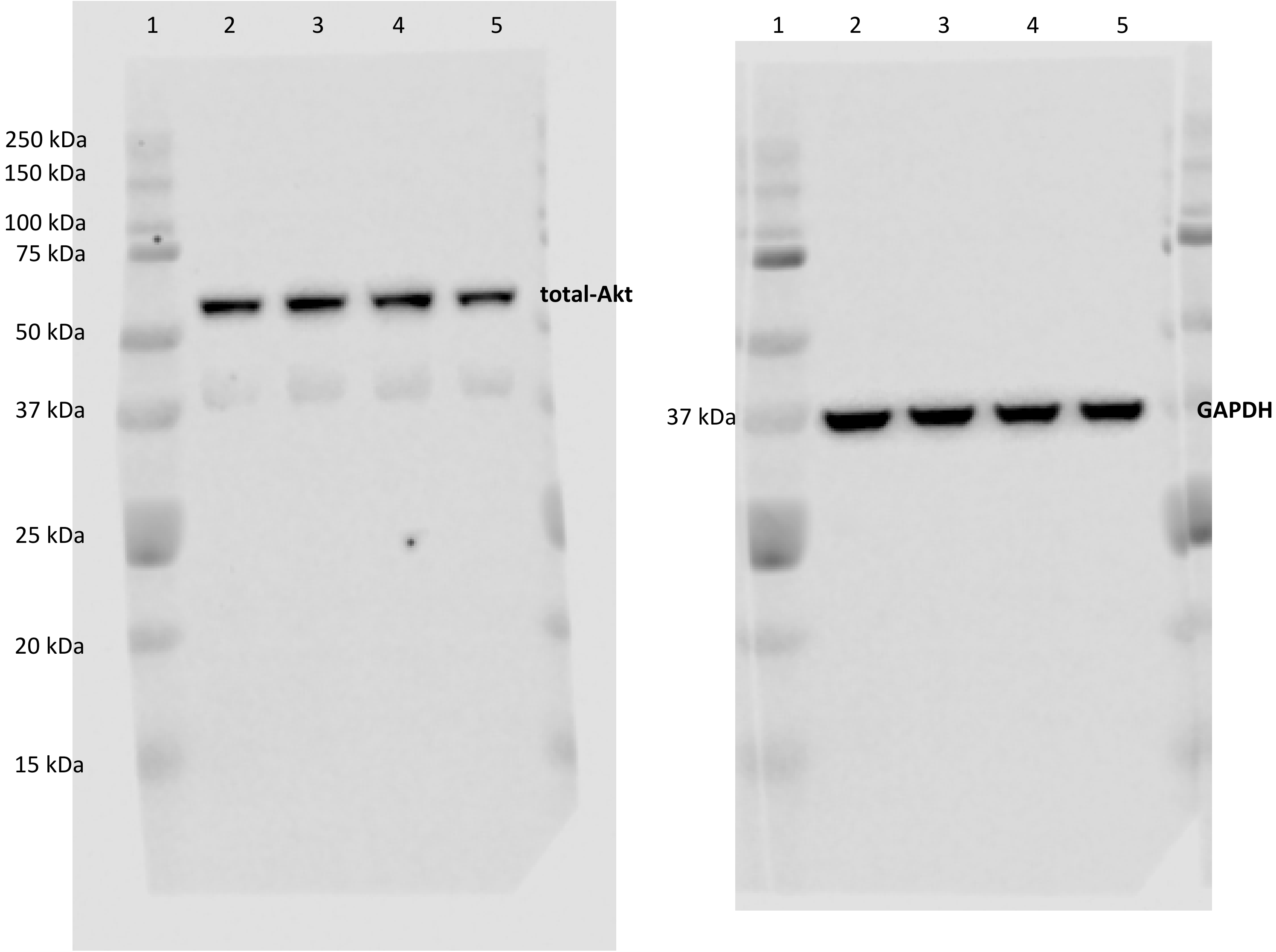

1 ladder

2 vehicle

3 2-AG

4 CXCL12

5 CXCL12+2-AG

JVM-2 cell line

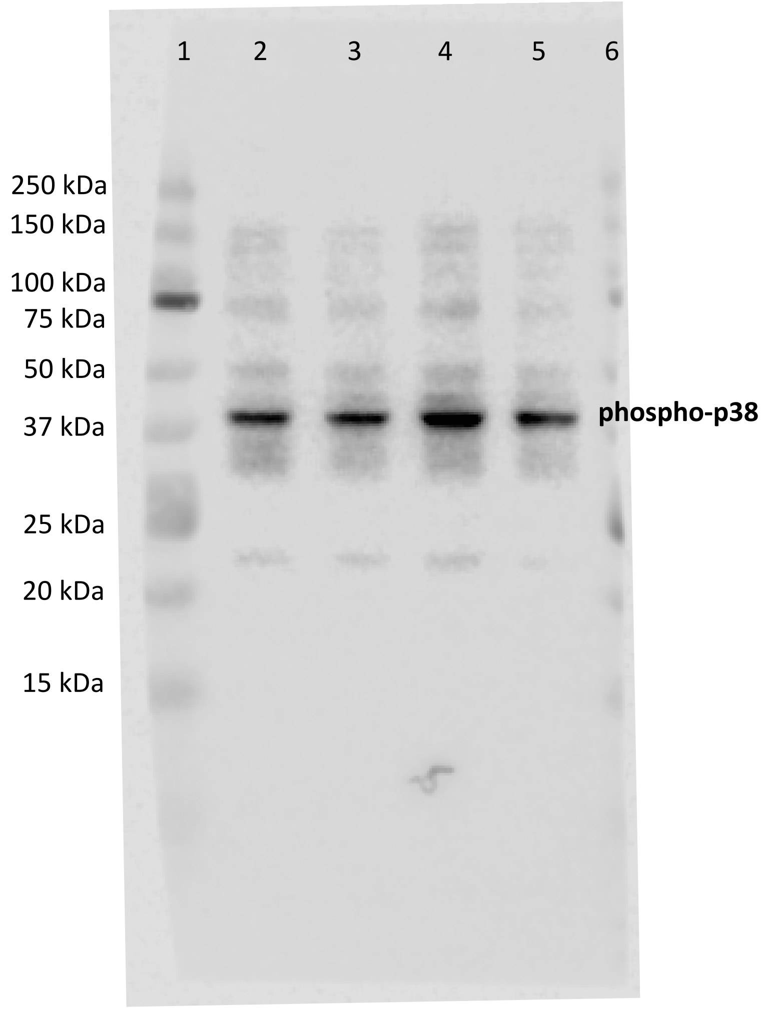

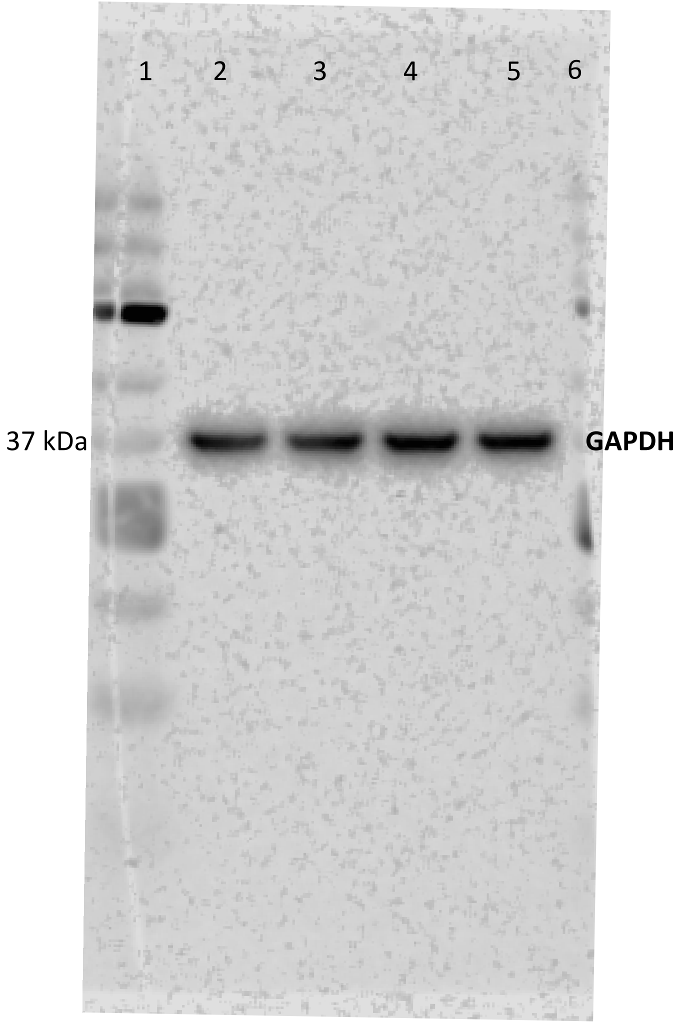

1 ladder

2 vehicle

3 2-AG

4 CXCL12

5 CXCL12+2-AG

6 ladder

JVM-2 cell line

JVM2 – Total-p38 – 20200905

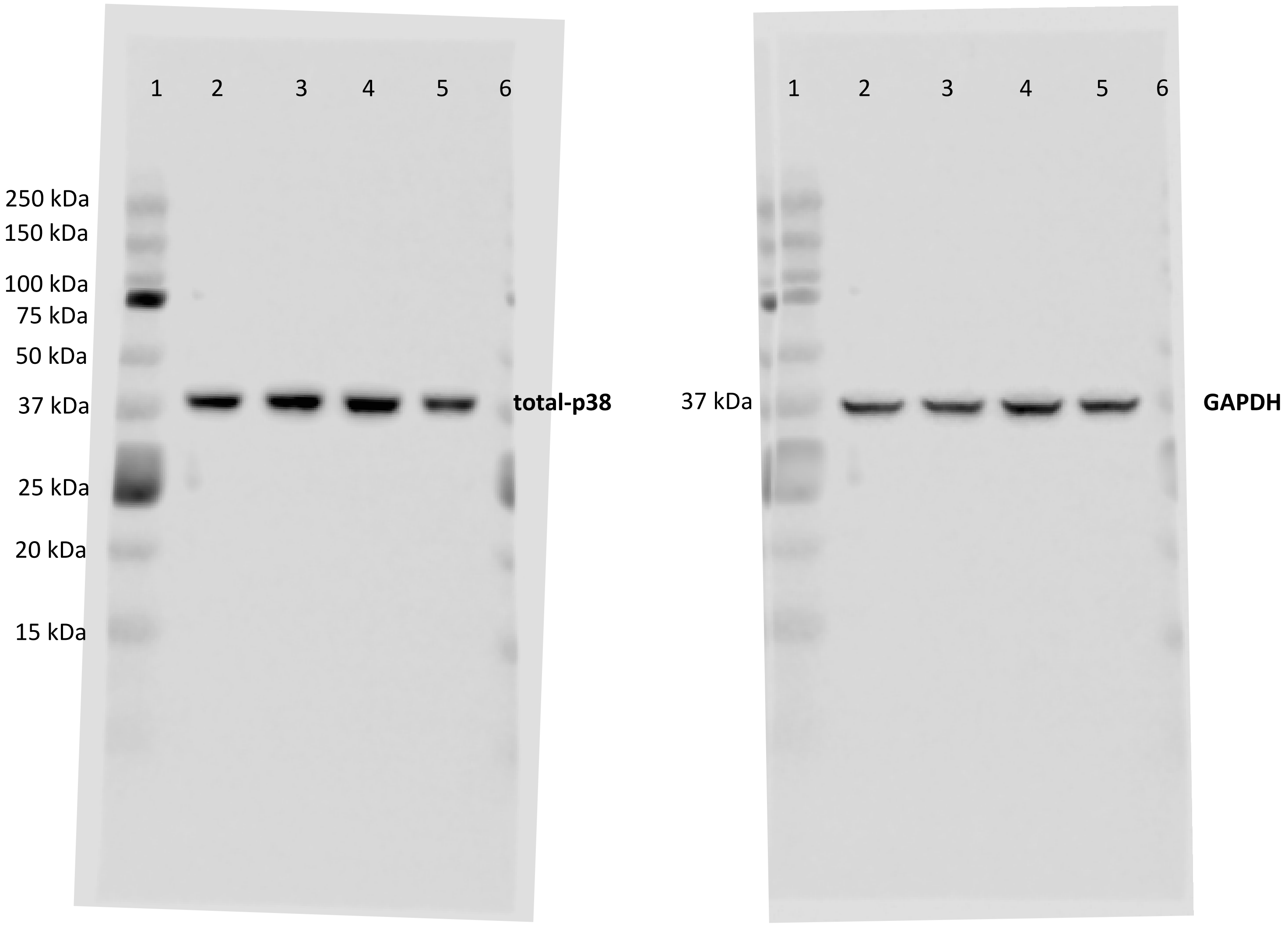

1 ladder

2 vehicle

3 2-AG

4 CXCL12

5 CXCL12+2-AG

6 ladder

Granta519 cell line

Granta519 – Phospho-ERK1/2 - 20210706

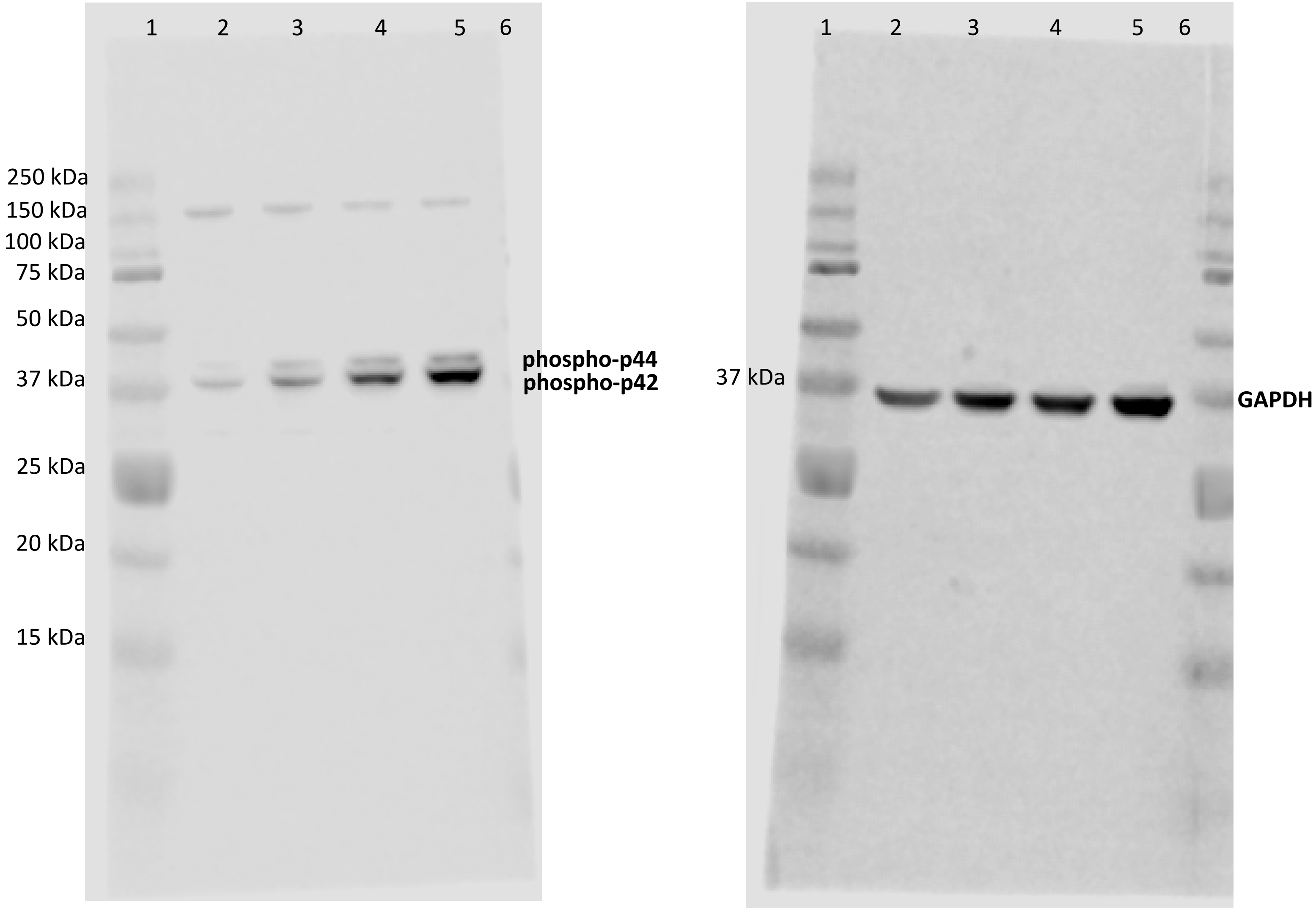

1 ladder

2 vehicle

3 2-AG

4 CXCL12

5 CXCL12+2-AG

6 ladder

Granta519 cell line

Granta519 – total-ERK1/2 - 20210706

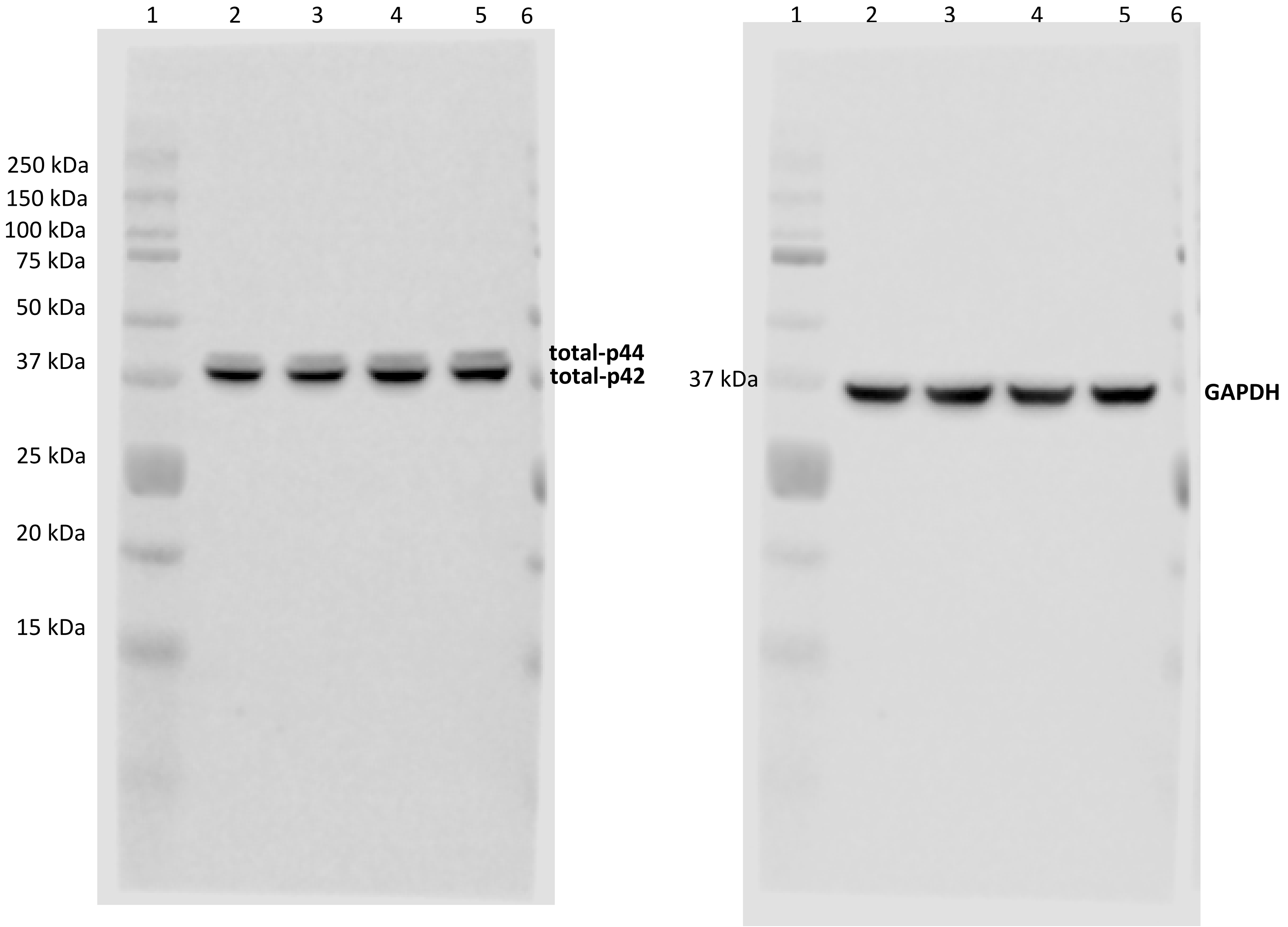

1 ladder

2 vehicle

3 2-AG

4 CXCL12

5 CXCL12+2-AG

6 ladder

Granta519 cell line

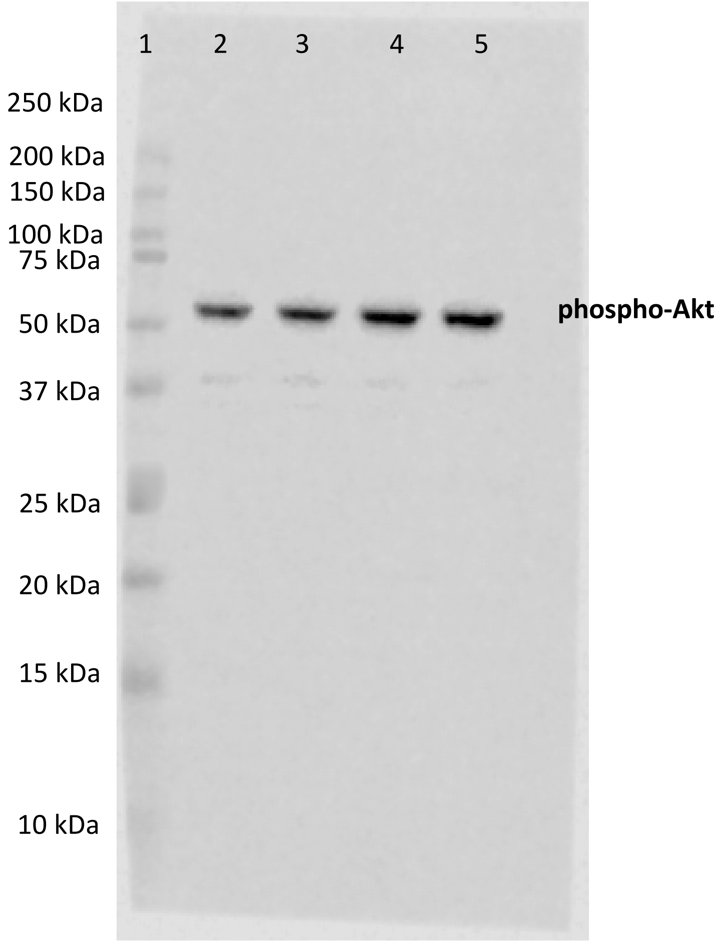

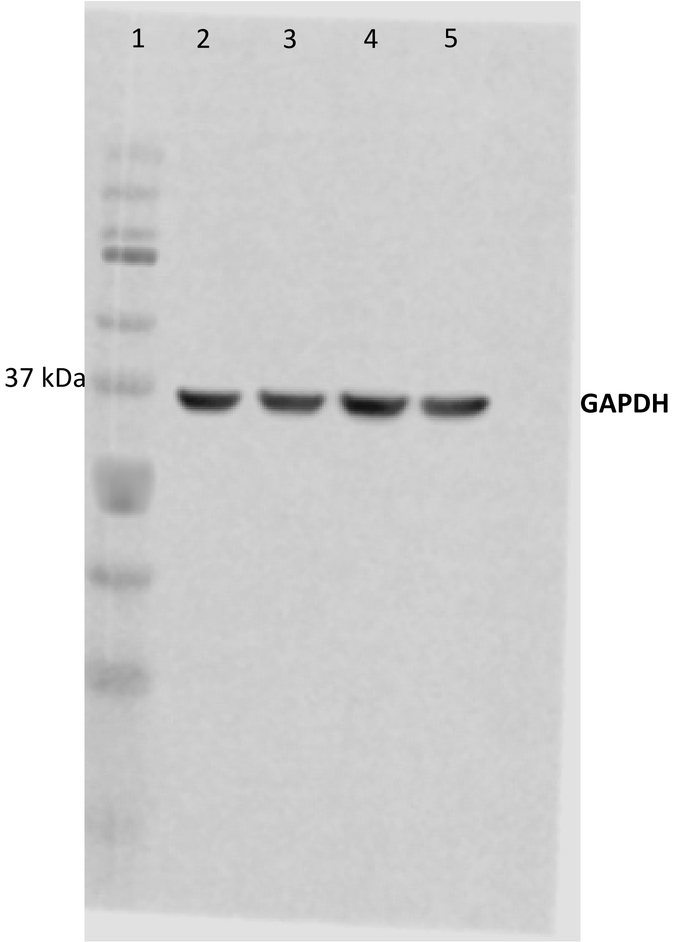

1 ladder

2 vehicle

3 2-AG

4 CXCL12

5 CXCL12+2-AG

Granta519 cell line

Granta519 – total-Akt- 20200705

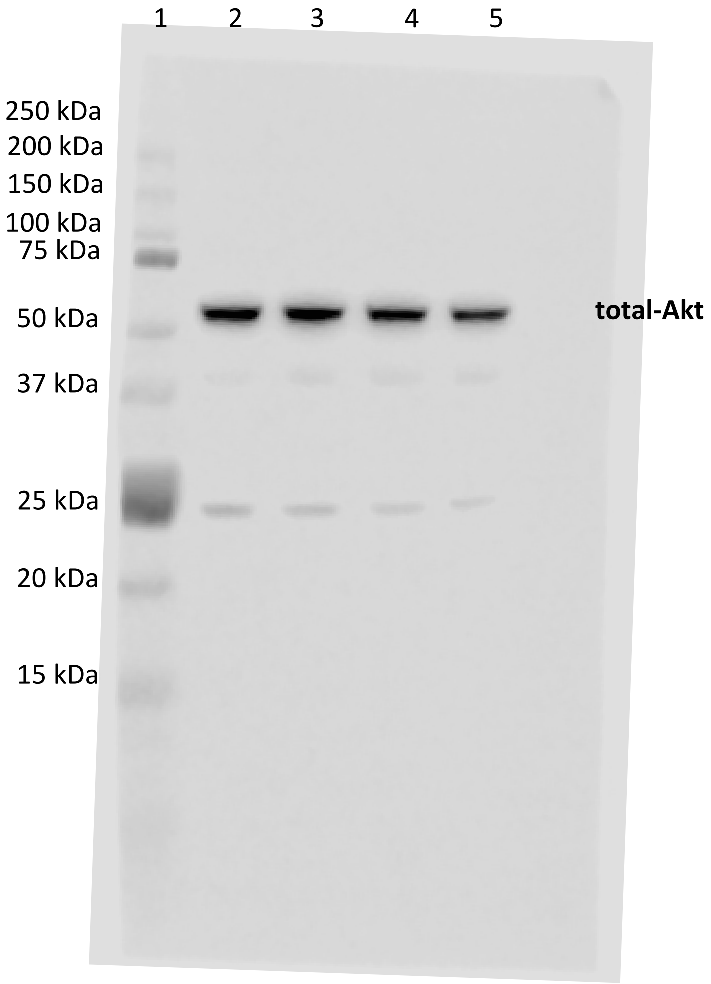

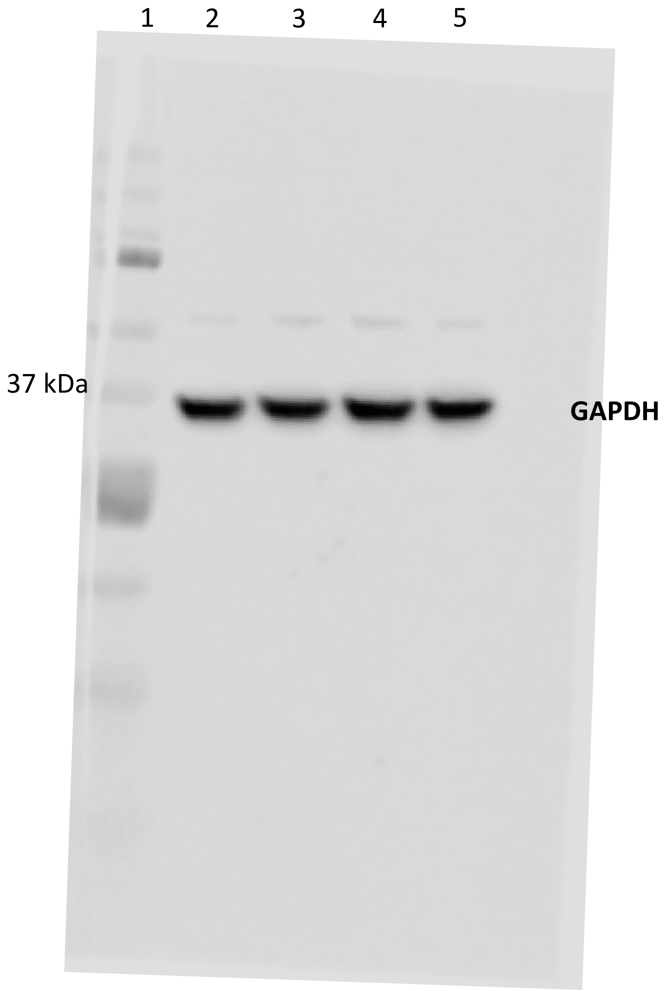

1 ladder

2 vehicle

3 2-AG

4 CXCL12

5 CXCL12+2-AG

Granta519 cell line

Granta519 – Phospho-p38 - 20210615

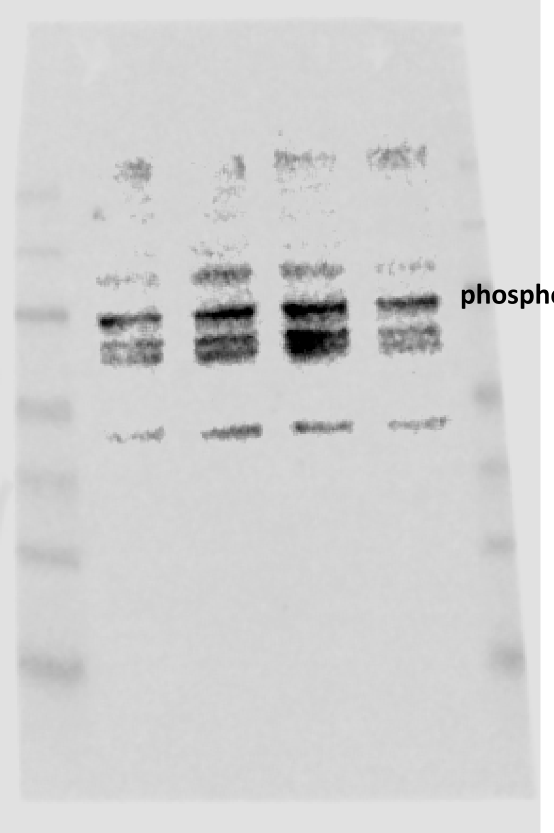

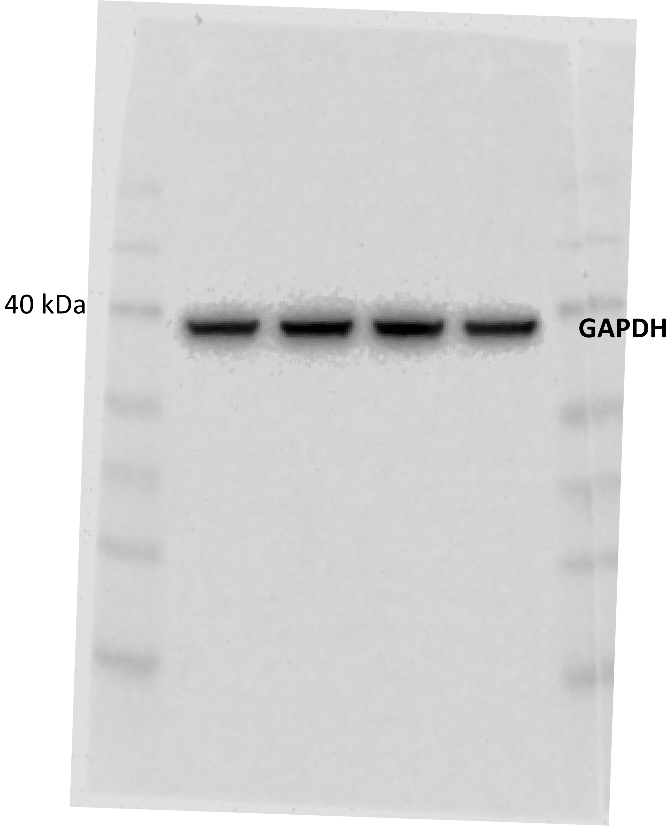

1 ladder

2 vehicle

3 2-AG

4 CXCL12

5 CXCL12+2-AG

6 ladder

Granta519 cell line

Granta519 – Total-p38 - 20210615

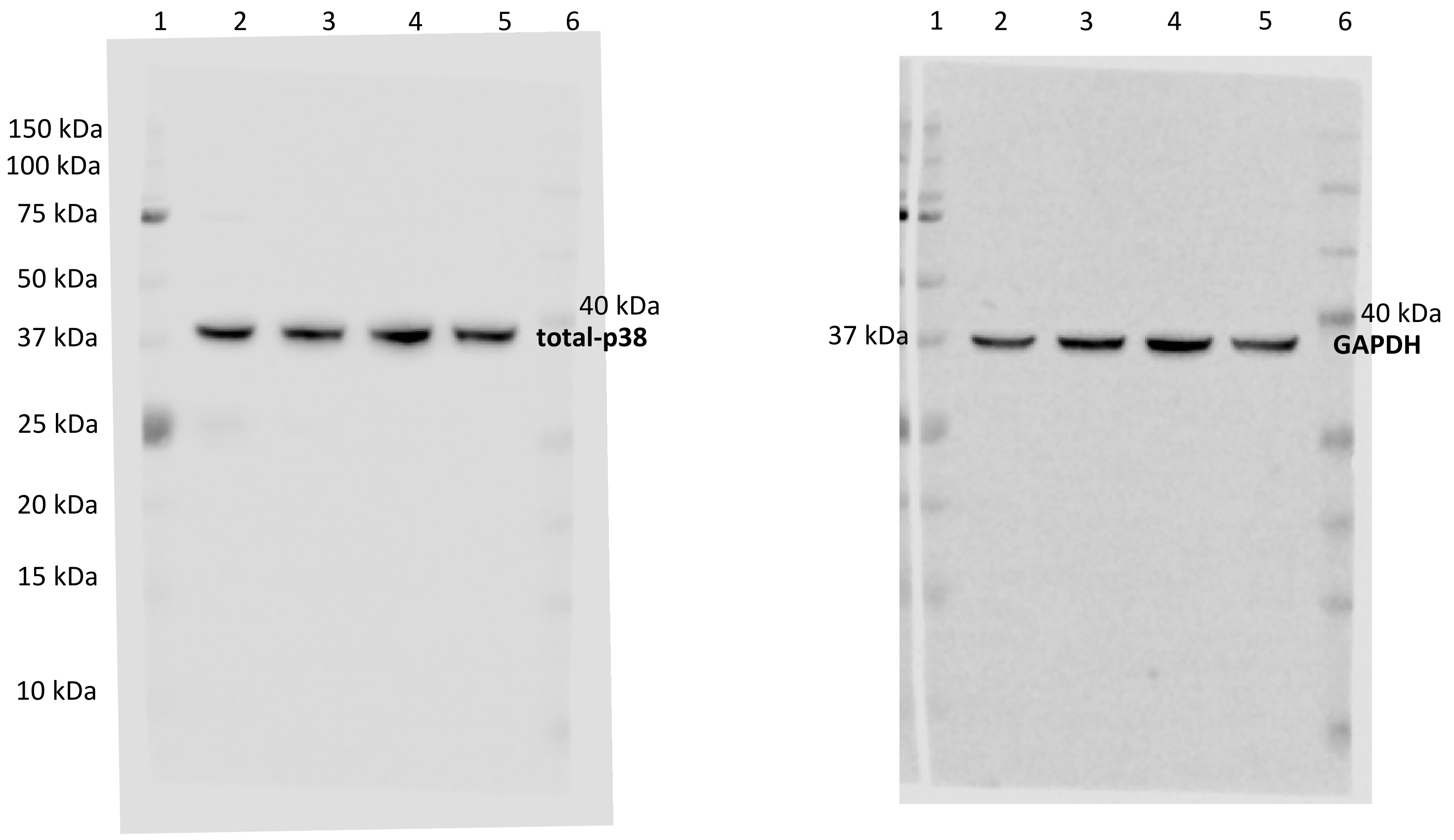

1 ladder

2 vehicle

3 2-AG

4 CXCL12

5 CXCL12+2-AG

6 ladder

JeKo-1 cell line

JeKo-1Phospho-ERK . Proteins 20200420- Blot 5May2020

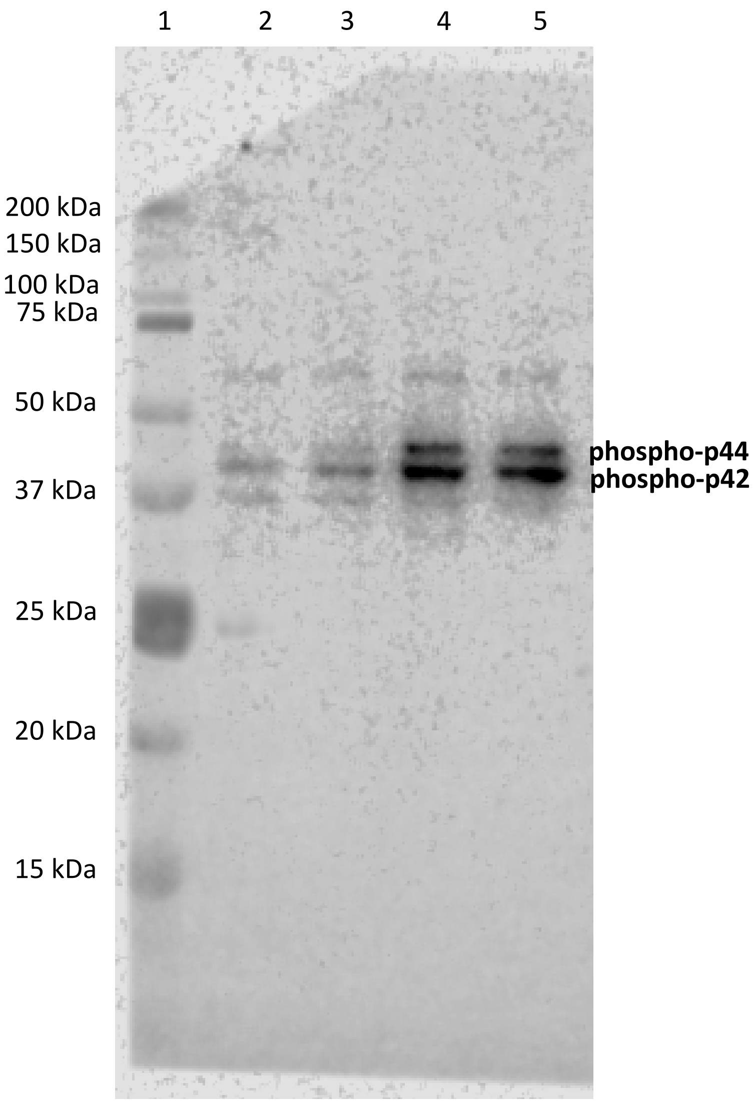

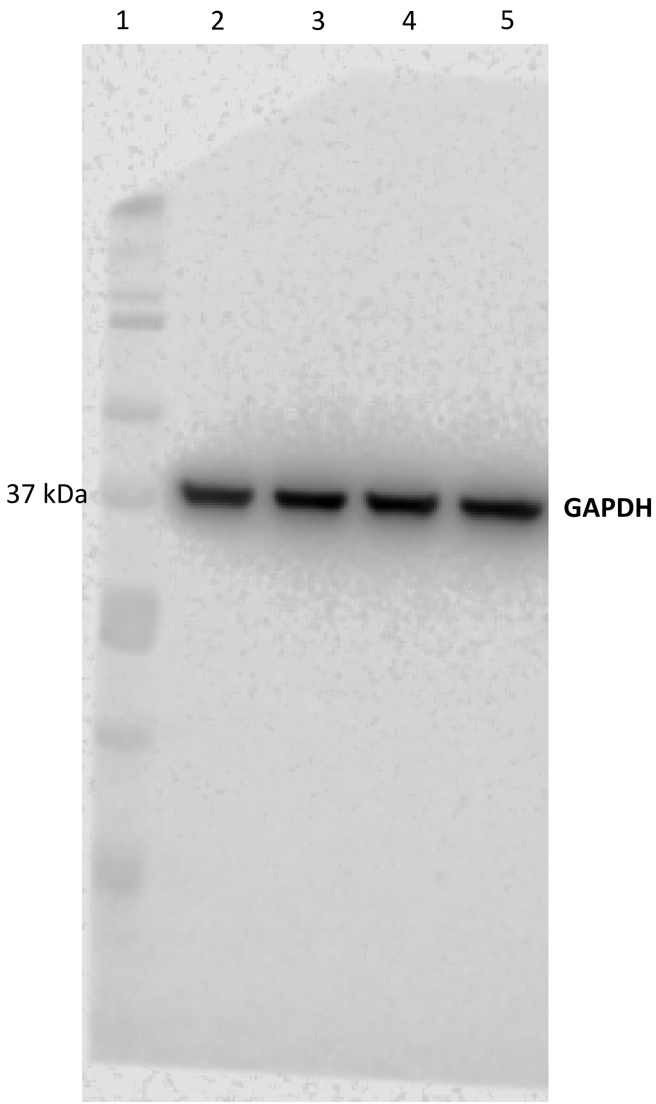

1 ladder

2 vehicle

3 2-AG

4 CXCL12

5 CXCL12+2-AG

JeKo-1 cell line

JeKo-1Total-ERK . Proteins 20200420- Blot 5May2020

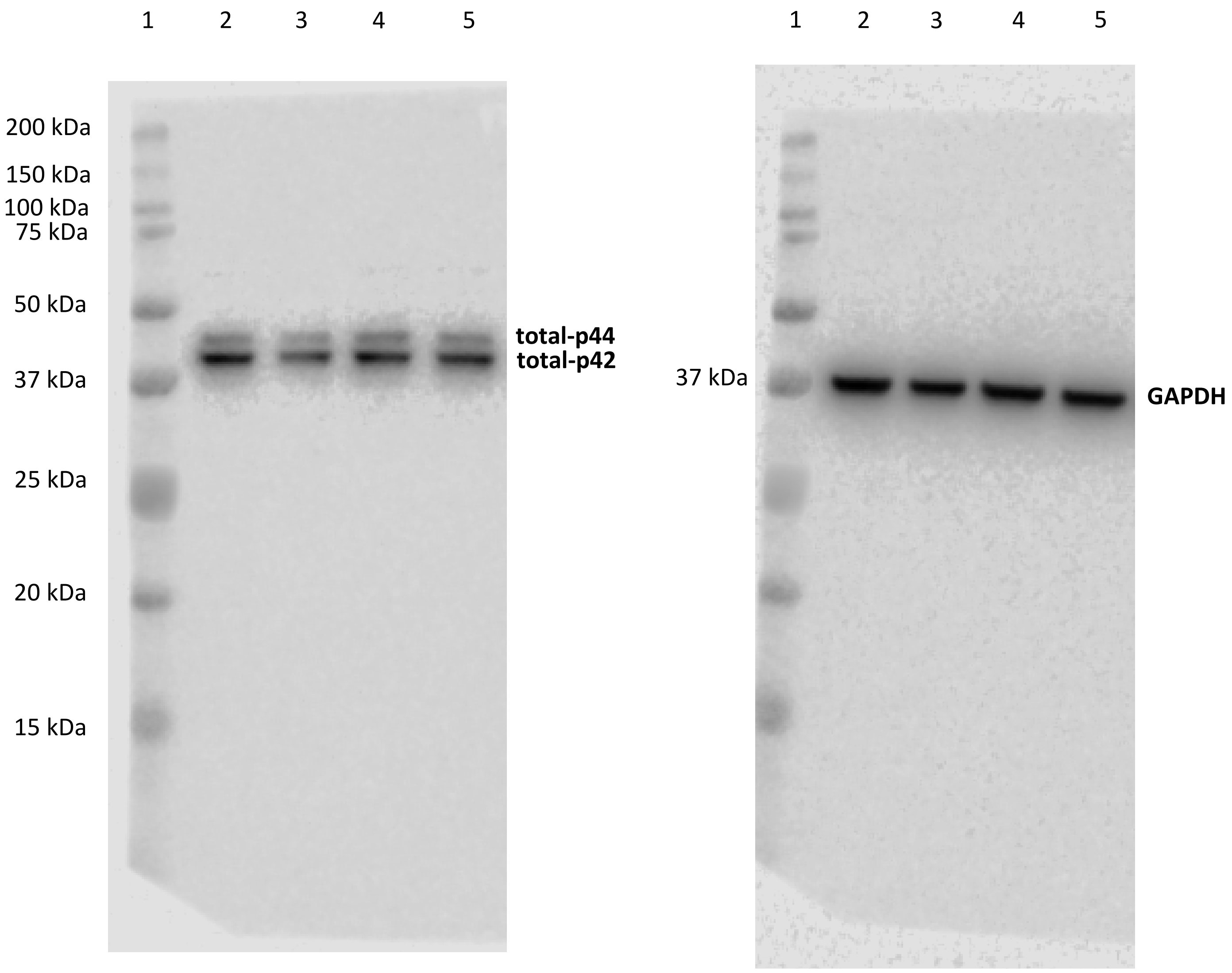

1 ladder

2 vehicle

3 2-AG

4 CXCL12

5 CXCL12+2-AG

JeKo-1 cell line

JeKo-1 – phospho-Akt- 20200430

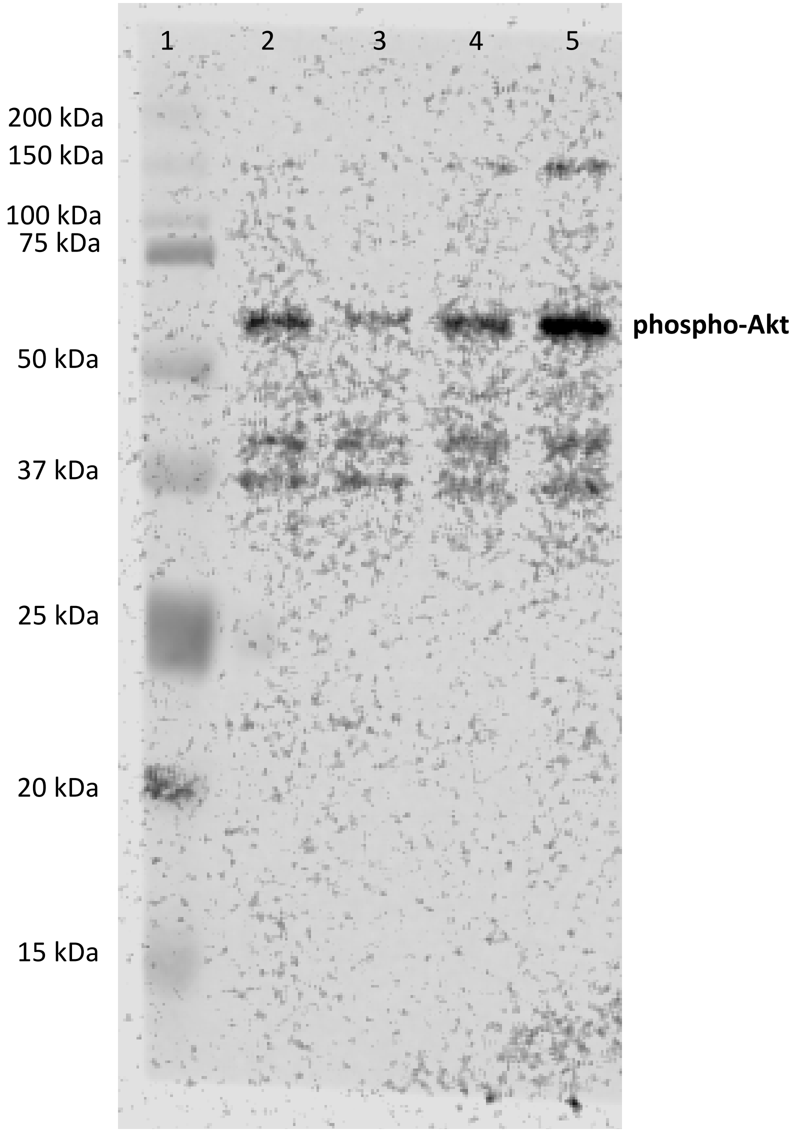

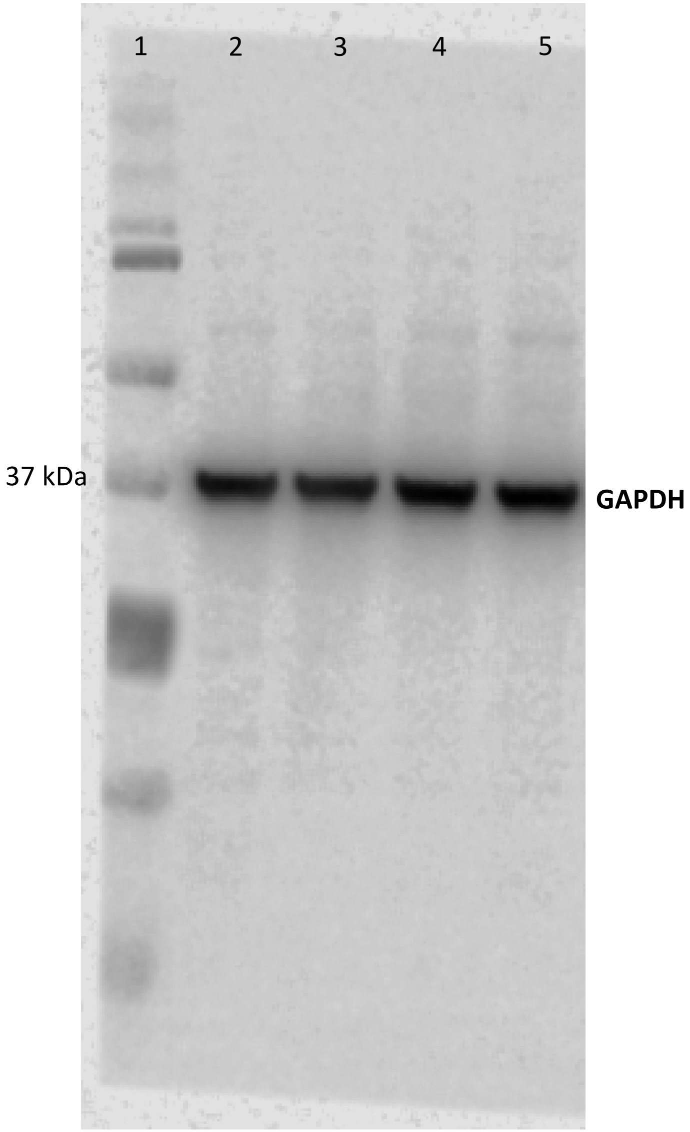

1 ladder

2 vehicle

3 2-AG

4 CXCL12

5 CXCL12+2-AG

JeKo-1 cell line

JeKo-1 – total-Akt- 20200430

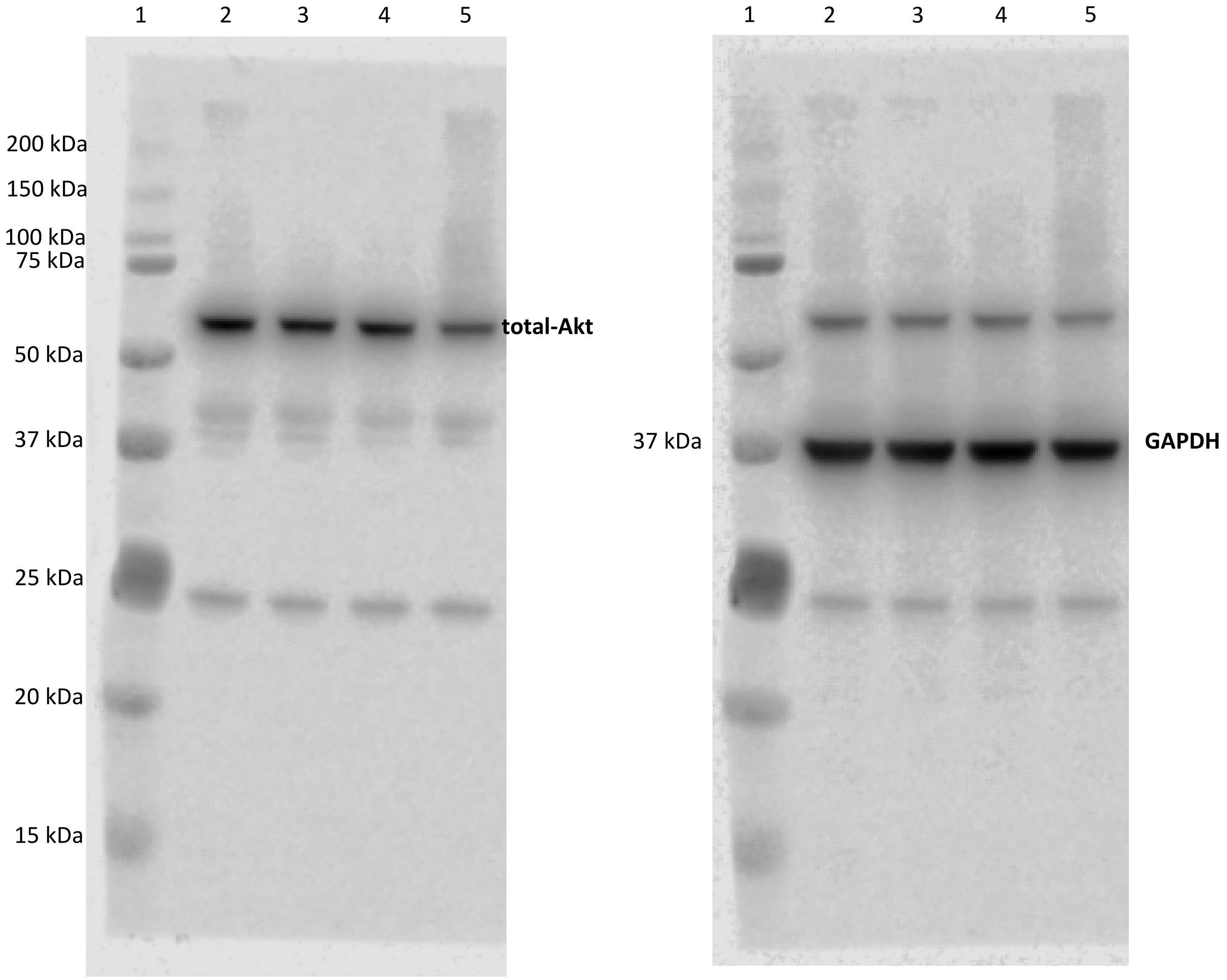

1 ladder

2 vehicle

3 2-AG

4 CXCL12

5 CXCL12+2-AG

JeKo-1 cell line

JeKo-1 Phospho-p38 - 20210922

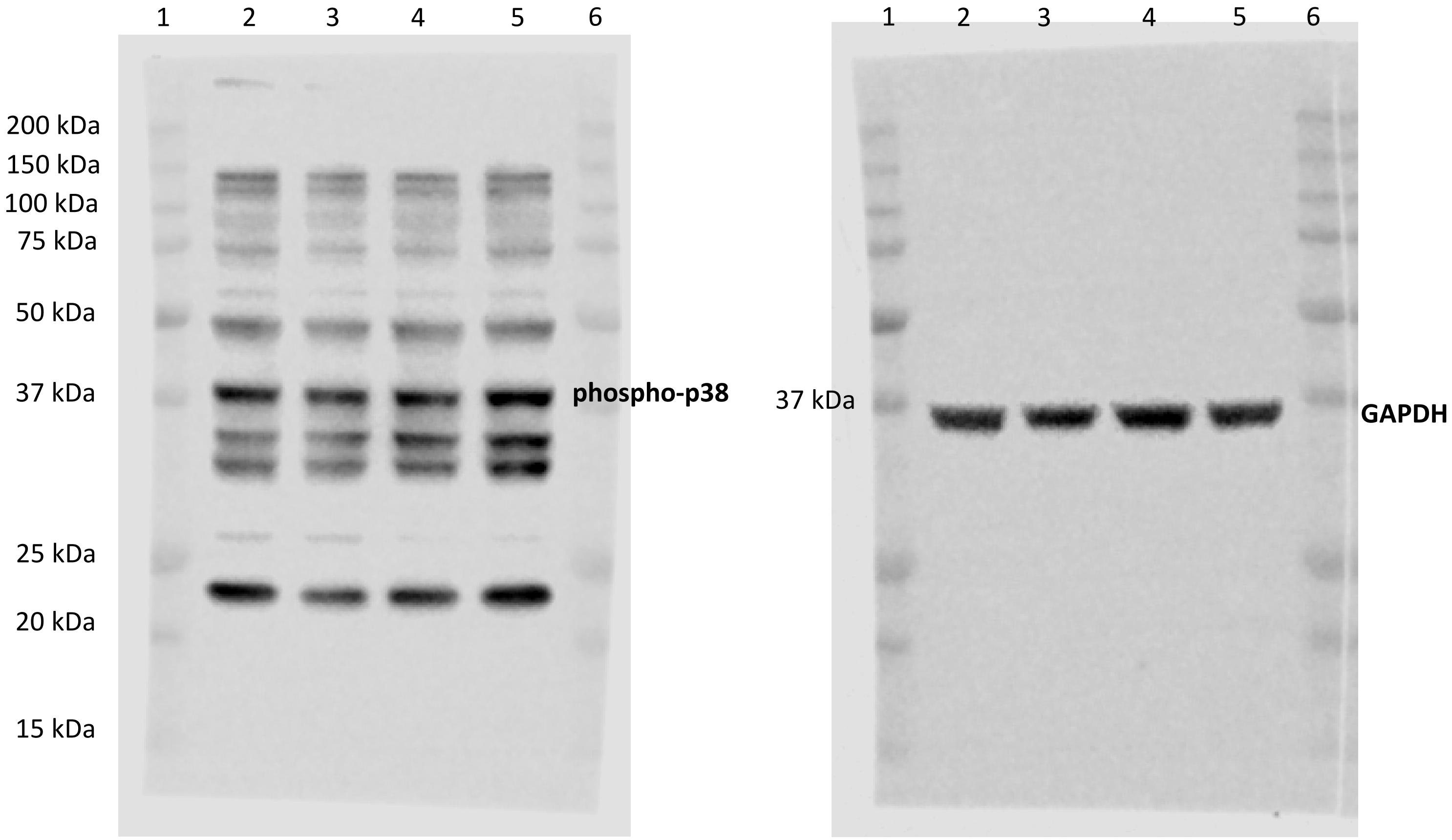

1 ladder

2 vehicle

3 2-AG

4 CXCL12

5 CXCL12+2-AG

6 ladder

JeKo-1 cell line

JeKo-1 Total-p38 - 20210922

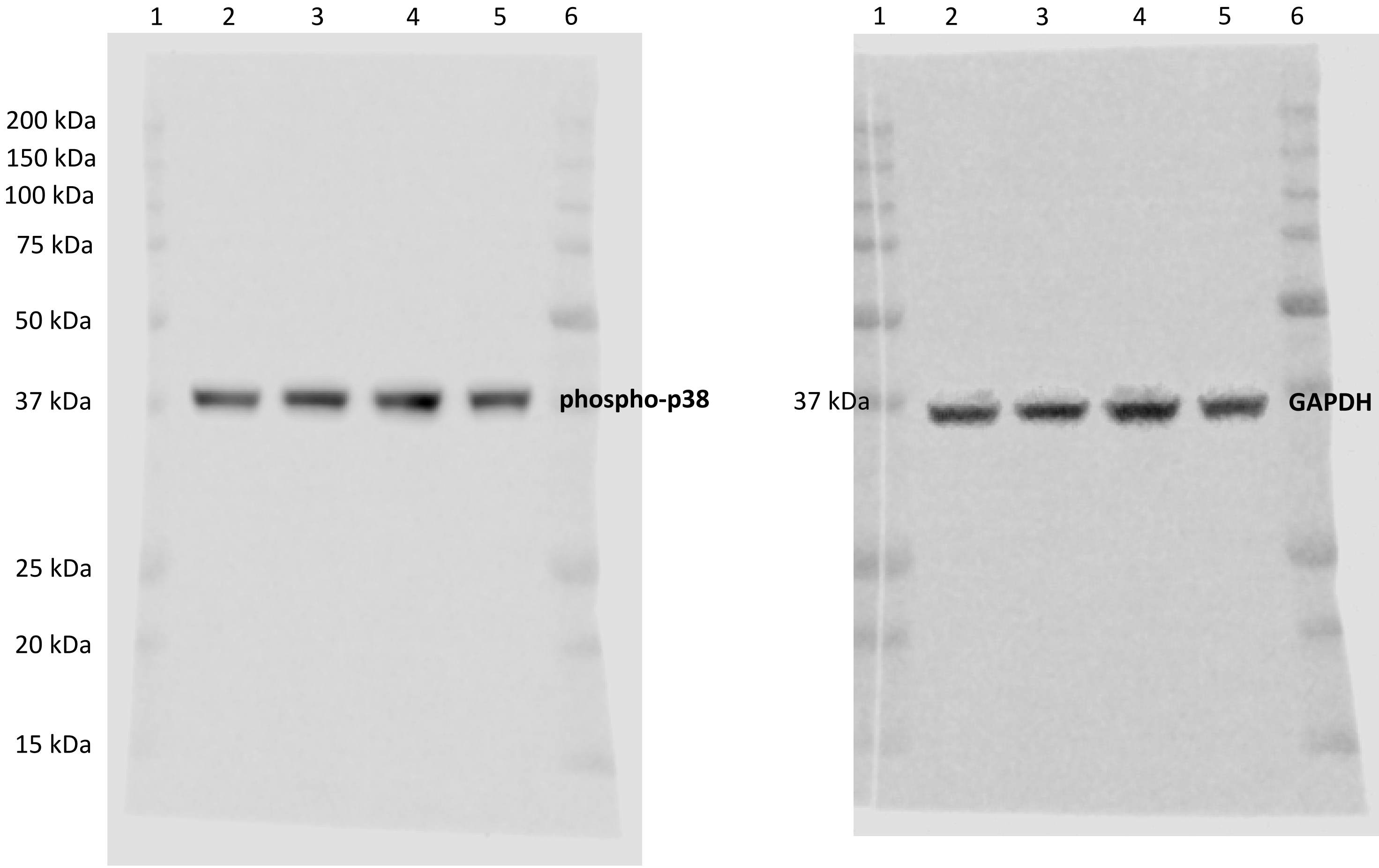

1 ladder

2 vehicle

3 2-AG

4 CXCL12

5 CXCL12+2-AG

6 ladder

Figure 9

Sample 40

Phospho-p38

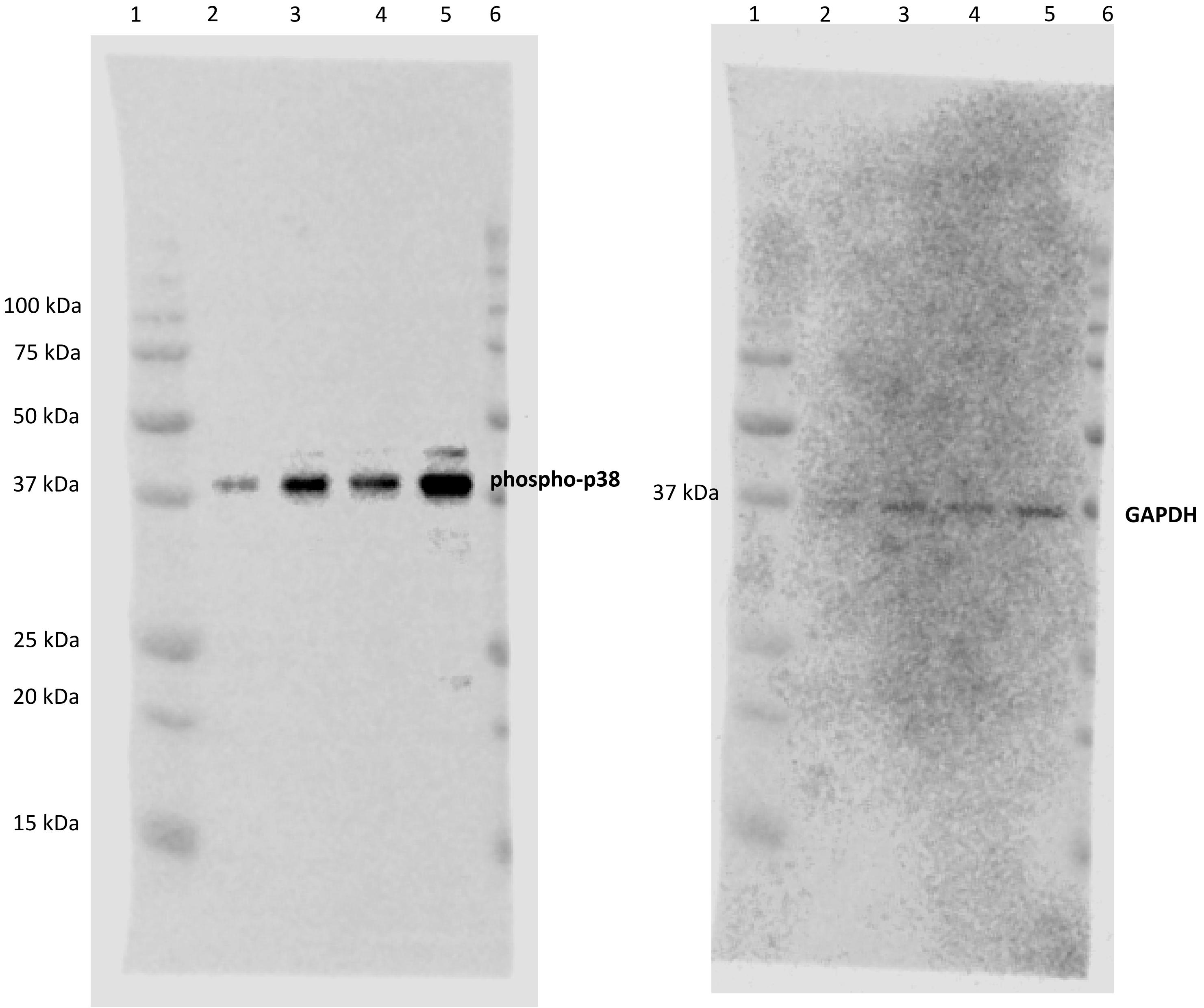

1 ladder

2 vehicle

3 2-AG

4 CXCL12

5 CXCL12+2-AG

6 ladder

Sample 40

Total-p38

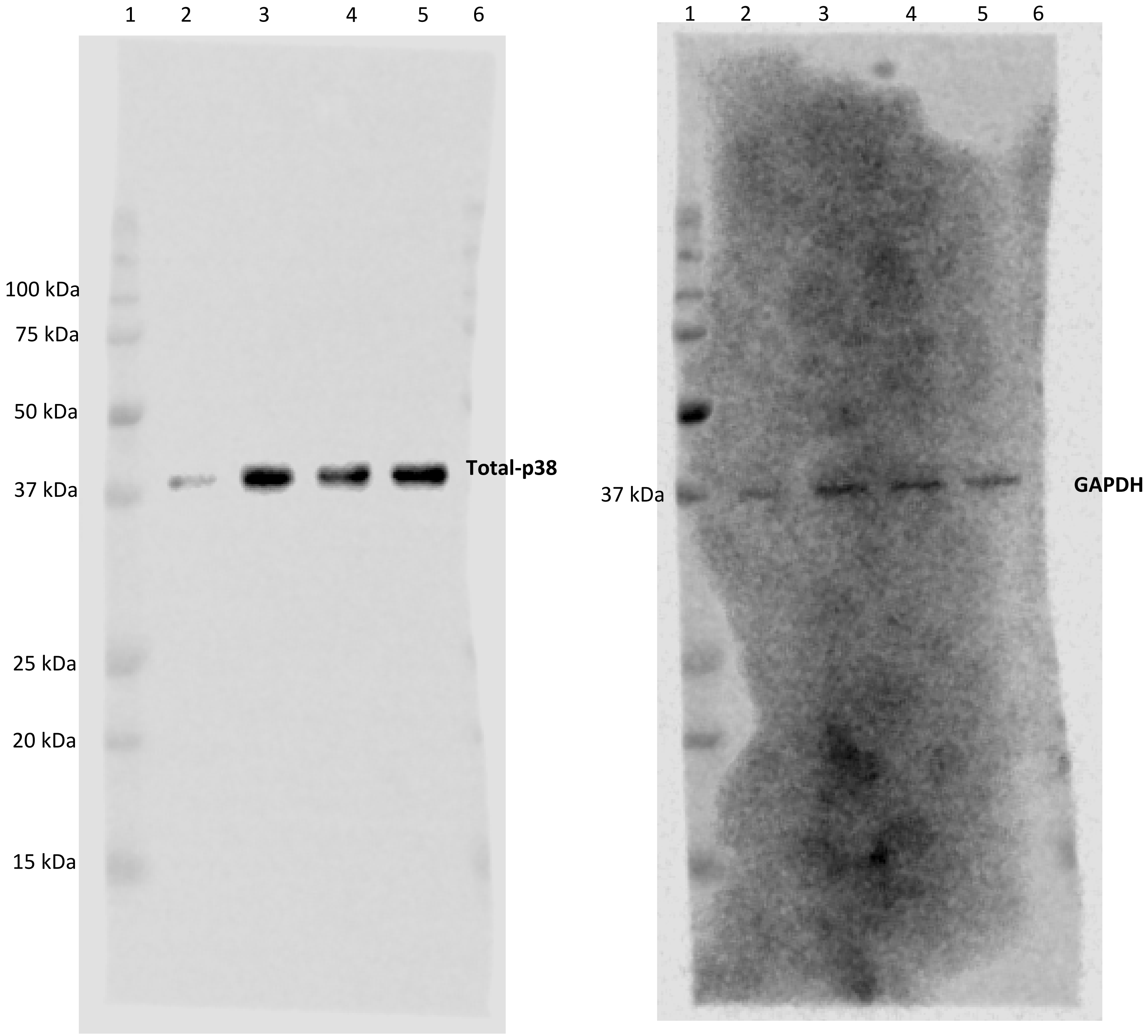

1 ladder

2 vehicle

3 2-AG

4 CXCL12

5 CXCL12+2-AG

6 ladder

Sample 40

Phospho-ERK1/2

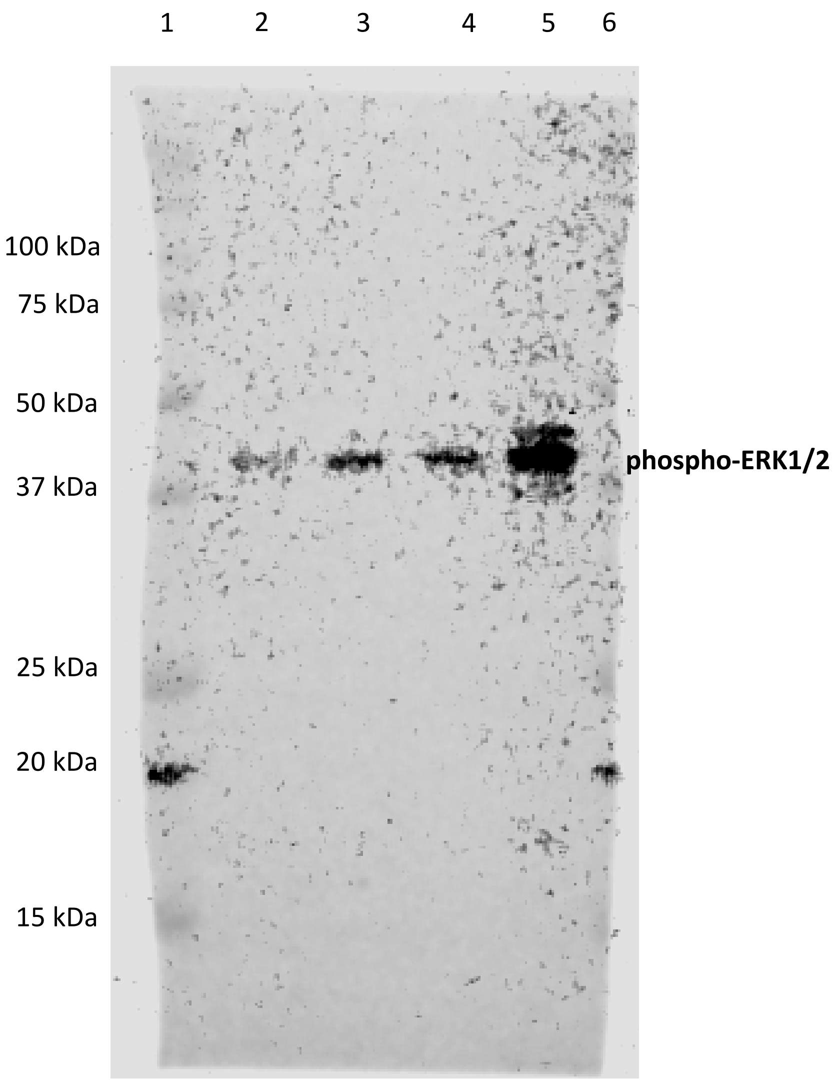

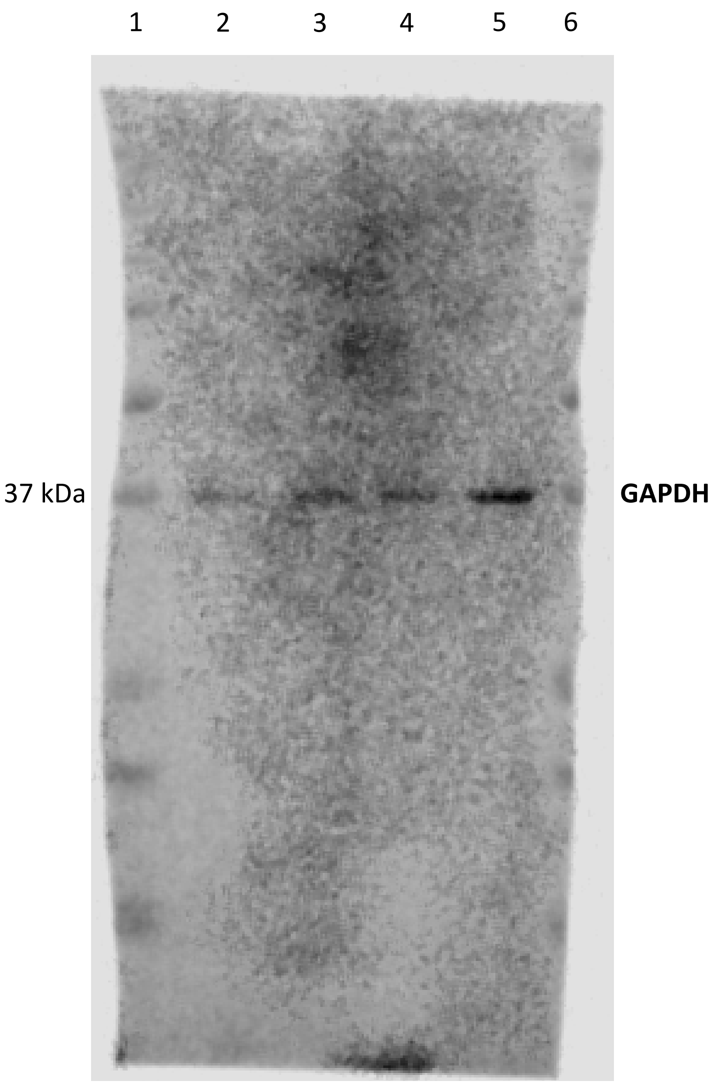

1 ladder

2 vehicle

3 2-AG

4 CXCL12

5 CXCL12+2-AG

6 ladder

Sample 40

Total-ERK1/2

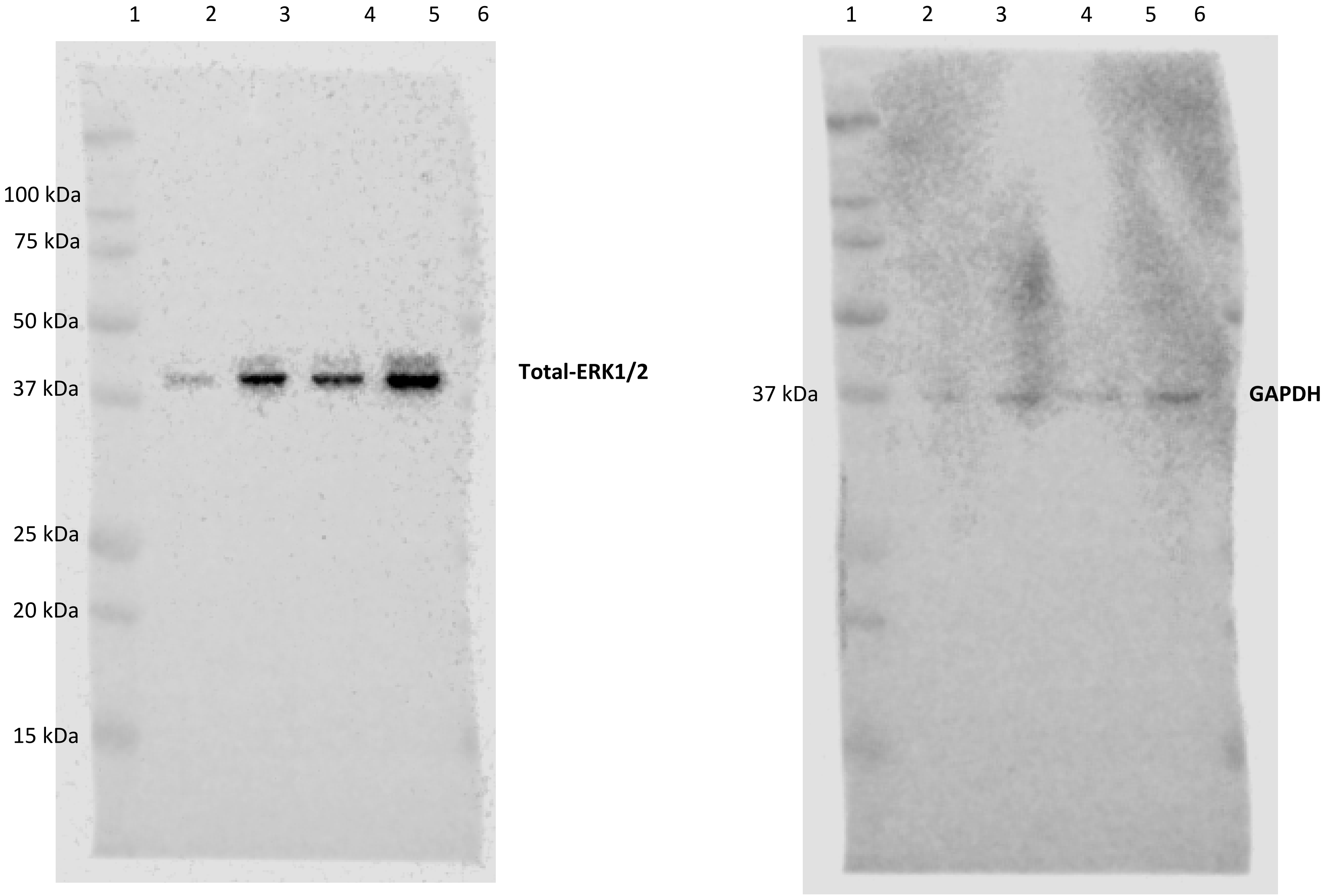

1 ladder

2 vehicle

3 2-AG

4 CXCL12

5 CXCL12+2-AG

6 ladder

Sample 41

Phospho-p38

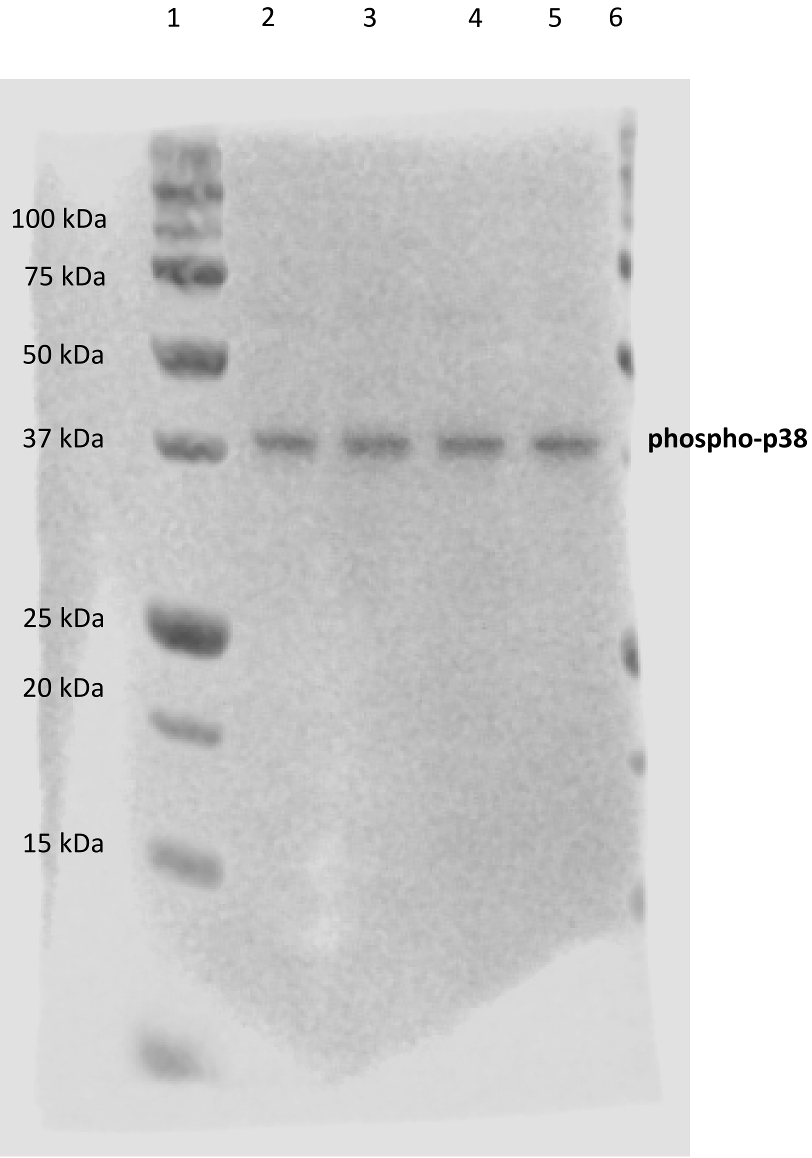

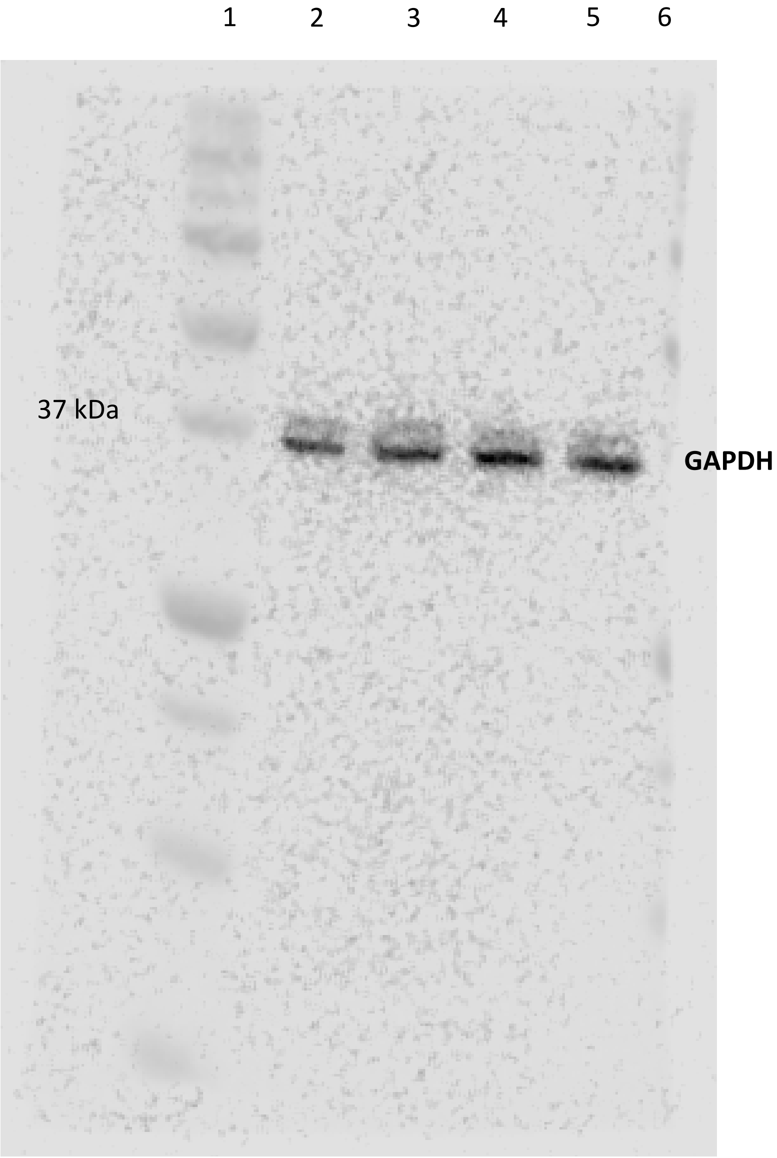

1 ladder

2 vehicle

3 2-AG

4 CXCL12

5 CXCL12+2-AG

6 ladder

Sample 41

Total-p38

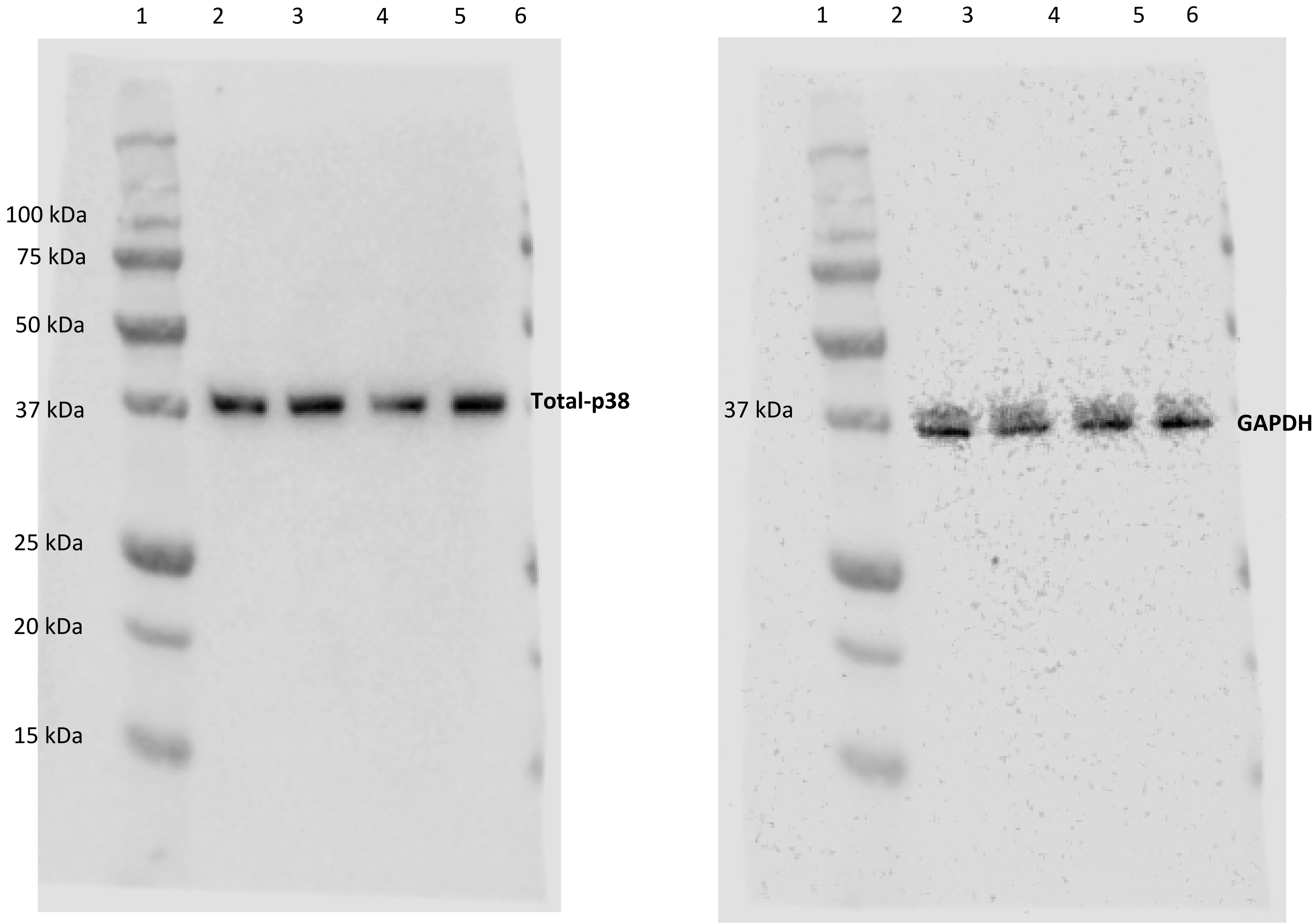

1 ladder

2 vehicle

3 2-AG

4 CXCL12

5 CXCL12+2-AG

6 ladder

Sample 41

Phospho-ERK1/2

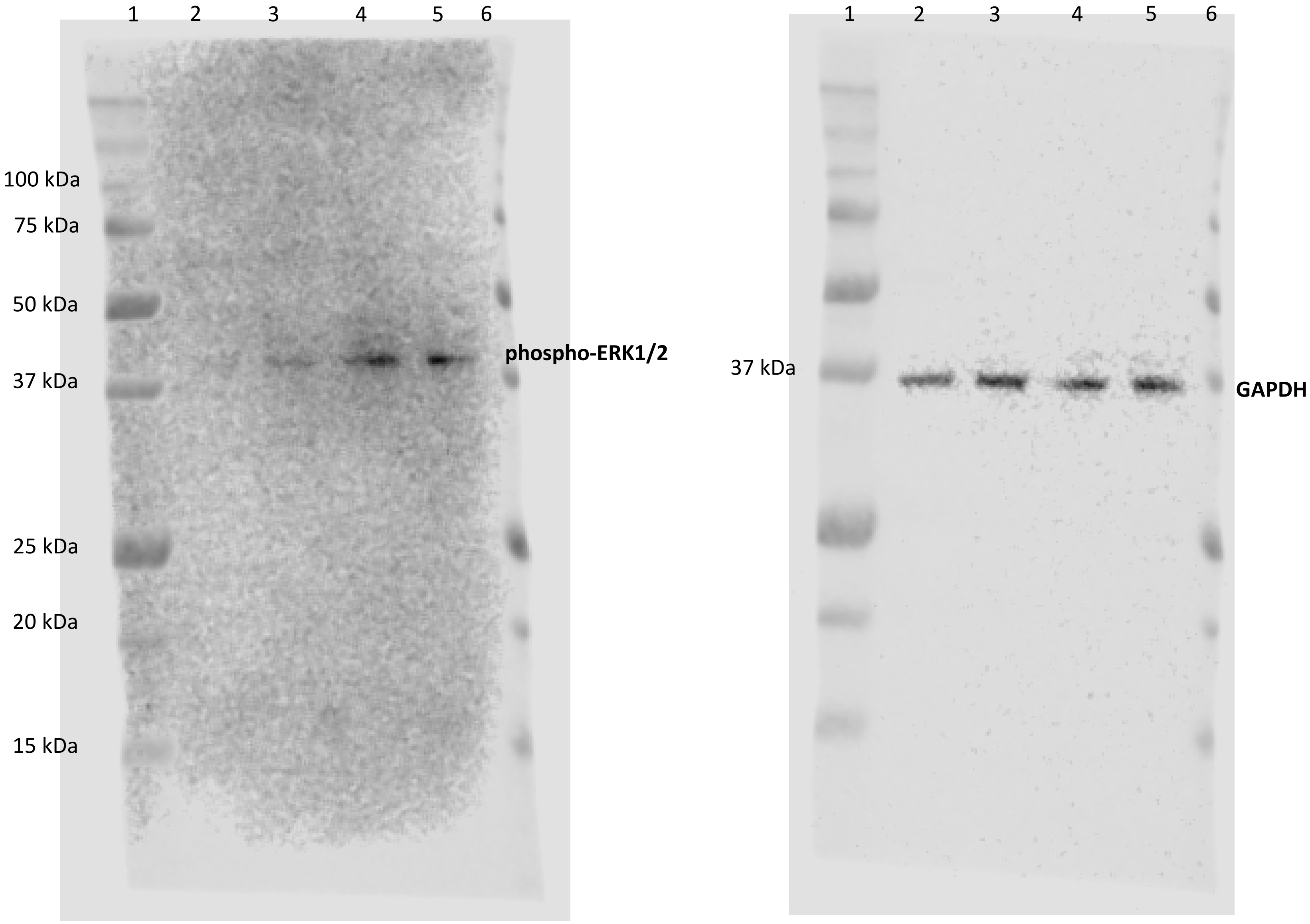

1 ladder

2 vehicle

3 2-AG

4 CXCL12

5 CXCL12+2-AG

6 ladder

Sample 41

Total-ERK1/2

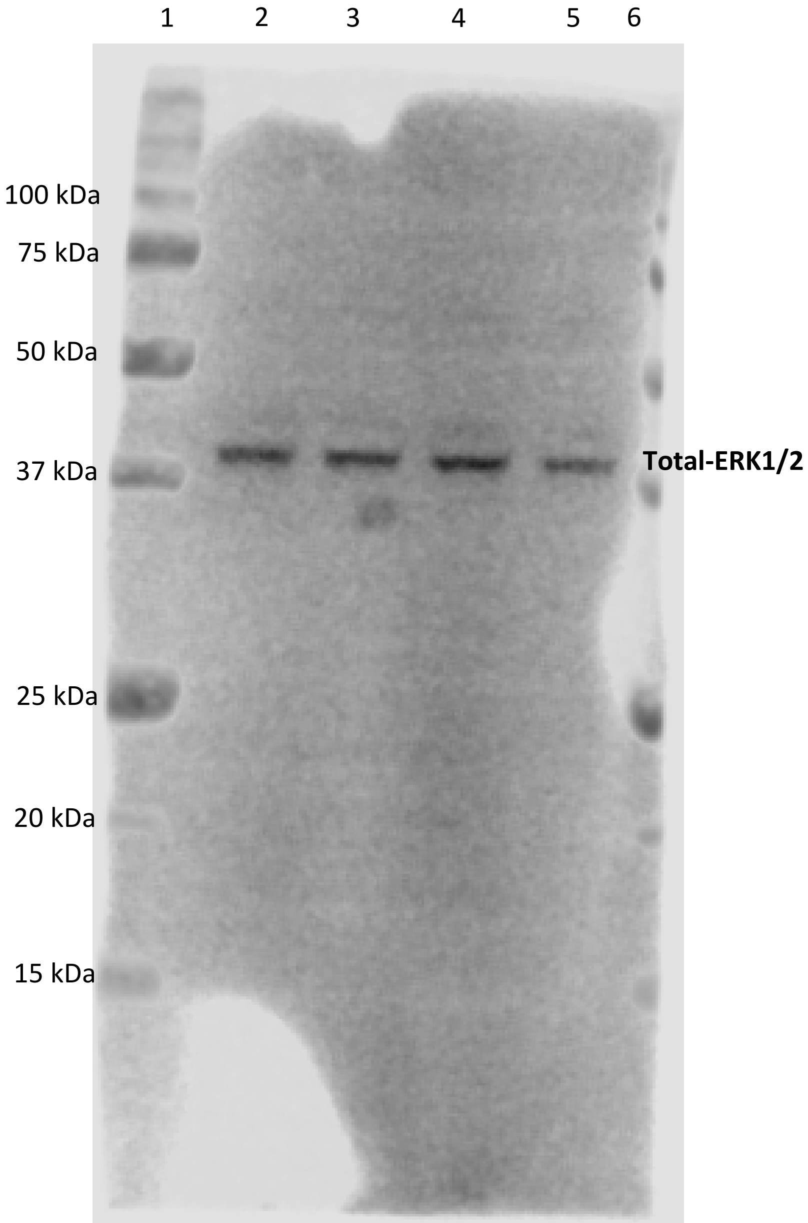

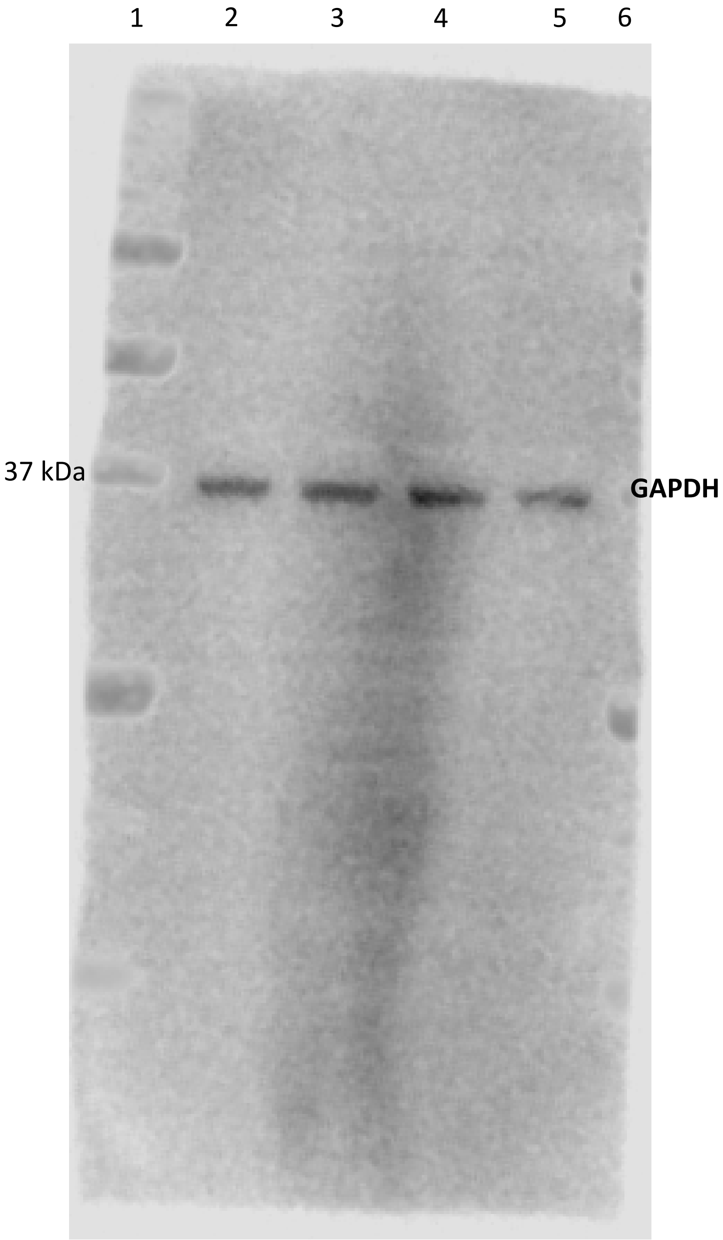

1 ladder

2 vehicle

3 2-AG

4 CXCL12

5 CXCL12+2-AG

6 ladder

Supplementary Figure S6

Cell lines

Cannabinoid receptors

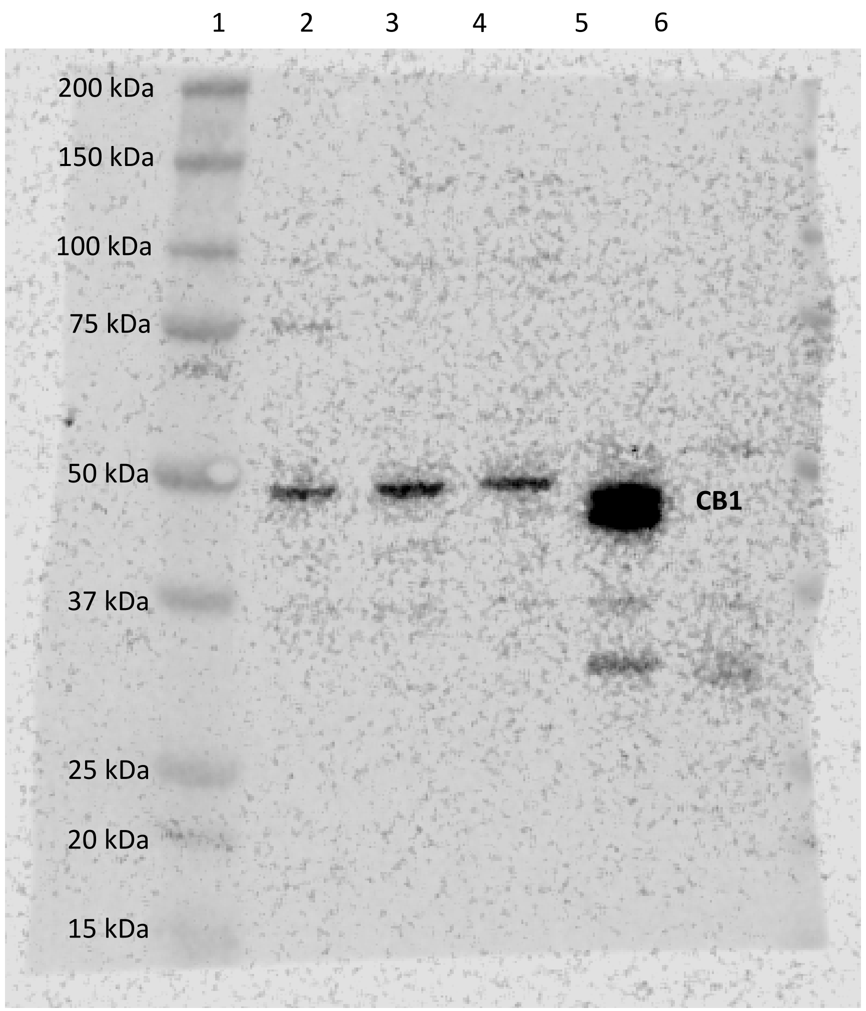

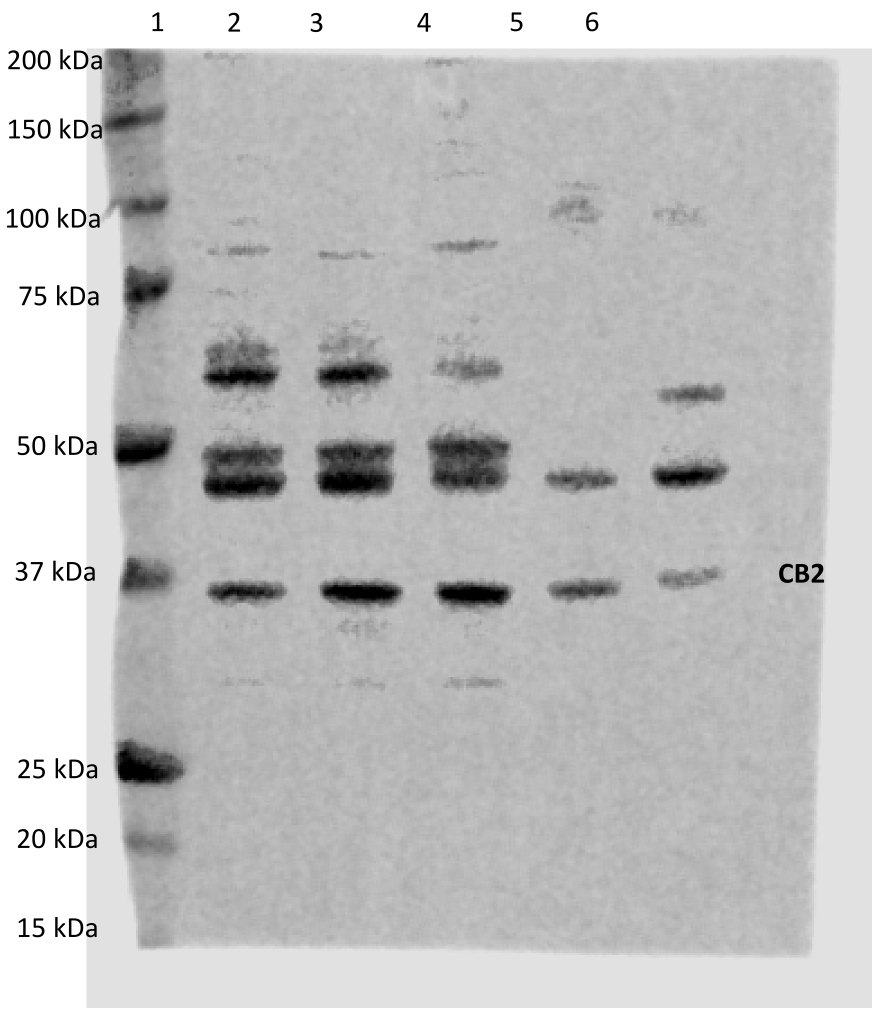

1 ladder

2 JVM-2

3 Jurkat

4 JeKo-1

5 Cortex
